# Microbiota‐derived 3‐Methyl‐L‐histidine mediates the proatherogenic effect of high chicken protein diet

**DOI:** 10.1002/mco2.70090

**Published:** 2025-02-13

**Authors:** Shanshan Zhu, Ludi Liu, Yawen Zhao, Bingqi Ye, Jialin He, Wenkang Li, Yingxi Xu, Jiangyuan Zhu, Min Xia, Yan Liu

**Affiliations:** ^1^ Guangdong Provincial Key Laboratory of Food, Nutrition and Health, and Department of Nutrition, School of Public Health Sun Yat‐sen University Guangzhou P. R. China; ^2^ Guangdong Provincial Key Laboratory of Food, Nutrition and Health, and Department of Statistics and Epidemiology, School of Public Health Sun Yat‐sen University Guangzhou P. R. China

**Keywords:** 3‐Methyl‐L‐hisitidine, atherosclerosis, gut microbiota, high chicken protein diet, intestinal cholesterol absorption

## Abstract

Diet rich in chicken protein has gained a widespread popularity for its profound effect on weight loss and glycemic control; however, its long‐term effect on cardiovascular health and the underlying mechanisms remains obscure. Here, we demonstrated that higher intake of chicken protein was an independent risk factor for sub‐clinical atherosclerosis. Adherence to high chicken protein diet (HCD) alleviated excessive weight gain and glycemic control regardless of the presence of gut microbiota in apolipoprotein E–deficient mice. In contrast, long‐term HCD administration enhanced intestinal cholesterol absorption and accelerated atherosclerotic plaque formation in a gut microbiota‐dependent manner. Integrative analysis of 16S rDNA sequencing and metabolomics profiling identified 3‐Methyl‐L‐histidine (3‐MH), resulting from an enrichment of *Lachnospiraceae*, as the key microbial effector to the atherogenic effect of HCD. Mechanistically, 3‐MH facilitated the binding of hepatocyte nuclear factor 1A (HNF1A) to the promoter of NPC1‐like intracellular cholesterol transporter 1 (NPC1L1), whereas inhibition of HNF1A–NPC1L1 axis abolished the atherogenic effect of 3‐MH. Our findings uncovered a novel link between microbiota‐derived 3‐MH and disturbed cholesterol homeostasis, which ultimately accelerated atherosclerosis, and argued against the recommendation of HCD as weight loss regimens considering its adverse role in vascular health.

## Graphical abstract

Schematic summary illustrates the potential mechanism, whereby high chicken protein diet promoted the progression of atherosclerosis. Long‐term adherence to a diet rich in chicken protein accelerated atherosclerosis through an enhanced microbial production of 3‐Methyl‐L‐histidine, which in turn facilitated NPC1L1‐mediated intestinal cholesterol absorption.

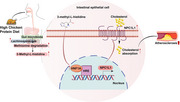

## 1 INTRODUCTION

Cardiovascular disease (CVD) remains the leading cause of global morbidity and mortality, affecting over 523 million individuals worldwide.^1^ Atherosclerotic cardiovascular diseases (ASCVDs) are primary contributors to the CVD burden.^2^ Optimal diet has been advocated as an effective and modifiable approach to reduce the burden of ASCVDs.^3^ Among various dietary regimes for the management of ASCVDs, diet rich in white meat protein has gained a widespread popularity for its profound effect on weight loss and improvement in glycemic control.^4^, ^5^ Due to its protective effect against obesity and its metabolic sequelae, the metabolic benefits of high‐white‐meat diet was assumed to extend to cardiovascular health. This notion was further supported by a meta‐analysis of observational studies showing that frequent intake of white meat was associated with a reduced risk for coronary mortality.^6^ However, a recent randomized controlled trial in generally healthy subjects showed that choosing white over red meat provided no additional benefits for reducing atherogenic lipoproteins, including low‐density lipoprotein cholesterol (LDL‐c) and apolipoprotein B.^7^ So far, no consensus has been reached on the effects of diet rich in white‐meat protein on cardiovascular health.

Dietary nutrients are essential not only for human health but also for maintaining the hemostasis of trillions of microbes residing in the intestine, the dysbiosis of which has recently been implicated in the progression of ASCVDs.^8^ A growing body of studies have provided strong evidence supporting the diet‐gut microbiota crosstalk on cardiovascular health in both positive and negative ways. On the one hand, a high‐fat diet increased the abundance of *Enterobacteriales*, resulting in enhanced gut permeability and systemic endotoxemia.^9^ Similarly, diet rich in red meat augmented the abundance of microbial *CutC/D* genes, leading to an increased biosynthesis of trimethylamine (TMA) followed by trimethylamine N‐oxide (TMAO).^10^ Mechanistically, TMAO accelerated atherosclerosis progression by facilitating foam cells formation, inducing platelet hyperreactivity and stimulating the release of inflammatory cytokines.^11^ On the other hand, fiber‐enriched diet and mediterranean diet have been linked to a higher richness and diversity of gut microbiota, and increased production of short‐chain fatty acids (SCFAs),^12^ which mainly acted through G‐protein receptors to reduce blood pressure and reverse aortic endothelial dysfunction.^13^ Collectively, microbial metabolites have been established as key molecular transducers of various diets to cardiovascular health. However, whether and how high white‐meat protein diet, a popular weight‐loss diet recently, modulates cardiovascular health through microbial metabolism remain largely unknown.

In this study, we demonstrated a pro‐atherogenic effect of high chicken protein diet (HCD) by showing that high intake of chicken protein, a common source of white‐meat protein globally, was associated with an increased likelihood of having carotid plaques, and that HCD exacerbated the progression of atherosclerosis in mice in a gut microbiota‐dependent manner. Moreover, we found that the atherogenic effect of HCD was attributed to an increased abundance of *Lachnospiraceae* and enhanced production of microbiome‐derived 3‐Methyl‐L‐hisdine (3‐MH), which in turn enhanced intestinal cholesterol absorption.

## 2 RESULTS

### 2.1 HCD‐modulated microbiota accelerates atherosclerotic plaque formation

**FIGURE 1:**
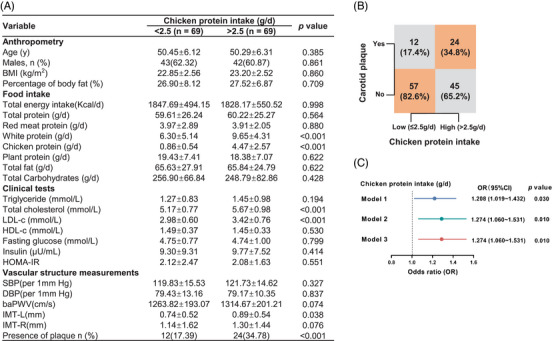
High intake of chicken protein was associated with increased risk of having carotid plaques. (A) Basic characteristics of the study participants. Data were presented as mean ± SD (*n* = 69 per group). (B) Proportion of subjects with carotid plaques according to daily intake of chicken protein. (C) Crude and adjusted odds ratio of having carotid plaque with higher consumption of chicken protein. Model 1: adjusted for none; Model 2: adjusted for BMI, diastolic blood pressure and total cholesterol; Model 3: Model 2 plus intake of red meat and plant protein.

To explore the effect of chicken protein intake on cardiovascular health in humans, 69 community residents with high daily intake of chicken protein, and another 69 individuals with low consumption of chicken protein matched for age, sex, red and plant protein intake, were analyzed (Figure S1). Compared to those with low intake of chicken protein, individuals with a higher intake demonstrated an adverse lipid profile and a higher prevalence of carotid plaques, with no obvious difference in obesity and insulin sensitivity (Figure 1A,B). Even after extensive adjustment for established risk factors for ASCVDs, as well as the intake of red meat and plant‐based protein, high intake of chicken protein remained an independent risk factor for sub‐clinical atherosclerosis (Figure 1C), suggesting that adherence to a diet rich in chicken protein might be harmful to cardiovascular health.

**FIGURE 2:**
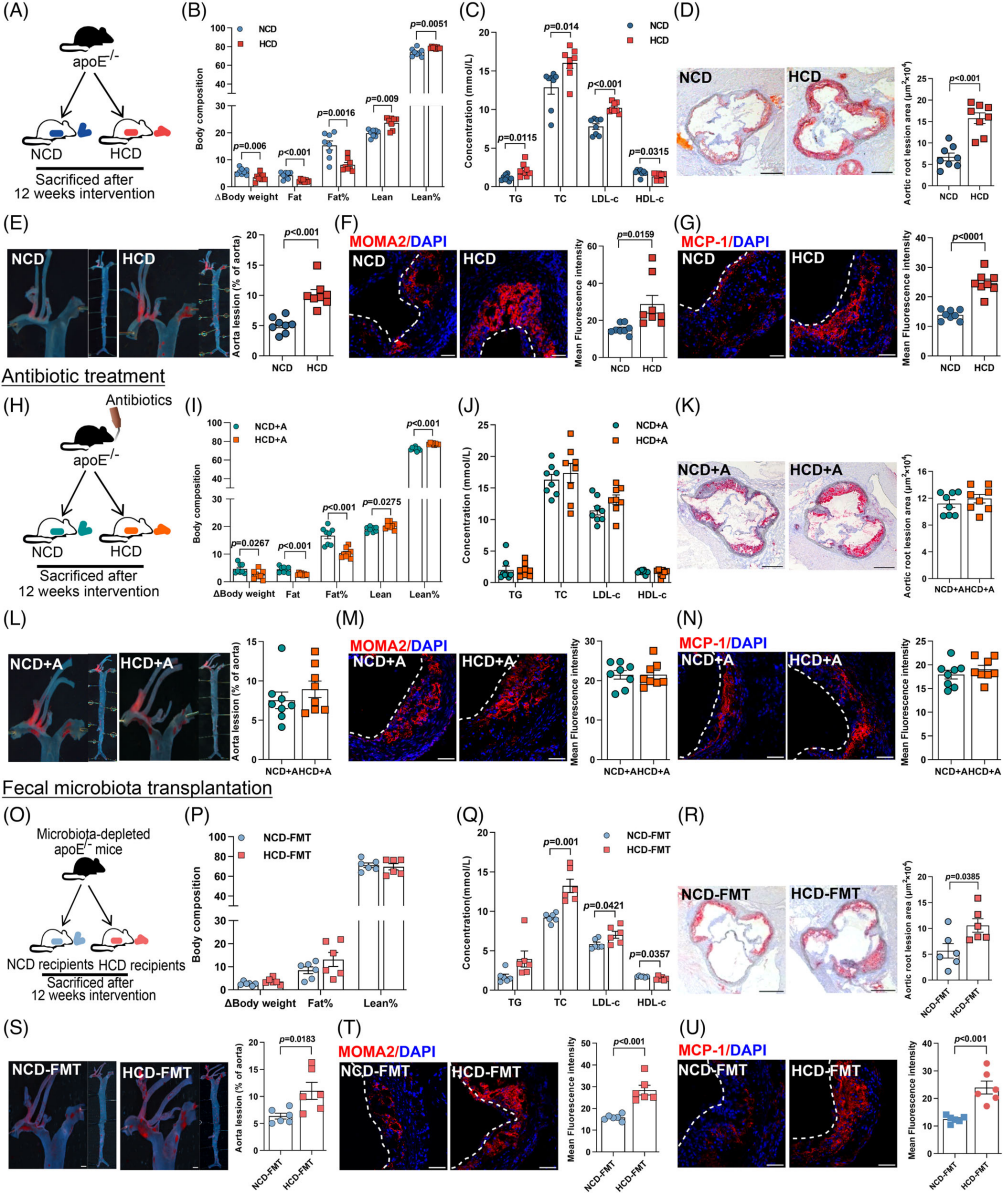
High chicken protein diet (HCD) exacerbated atherosclerotic plaque formation in a gut microbiota‐dependent manner. (A, H, and O) Experimental designs. (B, I, and P) Body compositions. (C, J, and Q) Serum levels of triglyceride, total cholesterol, low‐density lipoprotein cholesterol, and high‐density lipoprotein cholesterol. (D, K, and R) Cross‐sections of aortic sinuses stained with oil red O (scale bar represented 200 µm). (E, L, and S) En face staining of the entire aorta (scale bar represented 20 mm). (F, G, M, N and T, U) Macrophage infiltration and inflammation activation in aortic sinus determined by immunostaining for (F, M, and T) MOMA2 and (G, N, and U) MCP‐1, respectively (scale bar represented 50 µm). Data were represented as mean ± SEM (*n* = 8 per group for A–N, and *n* = 6 per group for O–U). Statistical analysis was performed by unpaired *t* test.

We then interrogated the pro‐atherogenic effect of HCD in apolipoprotein E (apoE^−/−^)‐deficient mice (Figure 2A, *n* = 8 per group). Though no difference in food intake (Figure S2A) was found, consistent with findings in subjects with metabolic syndrome,^14^ HCD intervention led to a substantial reduction in body weight gain and fat mass, accompanied by a modest but significant increase in lean mass (Figure 2B) and considerable improvement in glycemic control (Figure S2B–D). Given that ideal glycemic control not necessarily mean a reduction in cardiovascular risk,^15^ we next investigated the effect of HCD on cholesterol levels and plaque formation. In contrast to the beneficial effect on glucose metabolism, long‐term HCD administration augmented the levels of triglyceride, total cholesterol, and LDL‐c, but decreased HDL‐c (Figure 2C). Moreover, the plaque area in mice receiving HCD was almost three‐ and twofold that of those fed with normal chicken protein diet (NCD) in aortic root regions (Figure 2D) and the entire aorta (Figure 2E), respectively. Additionally, the increase in plaque area upon HCD intervention was accompanied by a marked increase in macrophage infiltration (Figure 2F) and an enhanced expression of monocyte chemoattractant protein‐1 (MCP‐1, Figure 2G), which was involved in promoting the adhesion of macrophages onto endothelium and subsequent transmigration into intima.^16^

To evaluate the potential involvement of gut microbiota, antibiotics cocktail was given to mice fed with NCD or HCD for 12 weeks (Figure 2H, *n* = 8 per group). Notably, with a relatively lower amount of food intake (Figure S2E), elimination of gut microbiota did not affect the beneficial effect of HCD on body composition (Figure 2I) and glucose metabolism (Figure S2F–H), whereas discrepancies in cholesterol levels (Figure 2J), plaque areas (Figure 2K,L), and local inflammation (Figure 2M,N) in mice exposed to NCD and HCD were abolished by sustained antibiotics treatment. To further elucidate the role of gut microbiota, we then administered fecal microbiota from mice fed with NCD or HCD to microbiota‐depleted apoE^−/−^ mice (Figure 2O, *n* = 6 per group). Mice receiving fecal microbiota from donors challenged with NCD or HCD demonstrated no significant difference in food intake (Figure S2I), body composition (Figure 2P), and glycemic control (Figure S2J‐L), but a substantial increase in circulating cholesterol levels (Figure 2Q), plaque areas (Figure 2R,S), and inflammation infiltration in the plaque areas (Figure 2T,U). Collectively, these findings demonstrated that HCD accelerated the progression of atherosclerosis in a gut microbiota‐dependent manner.

### 2.2 HCD promotes atherosclerotic plaque formation via enhancing intestinal cholesterol absorption

**FIGURE 3:**
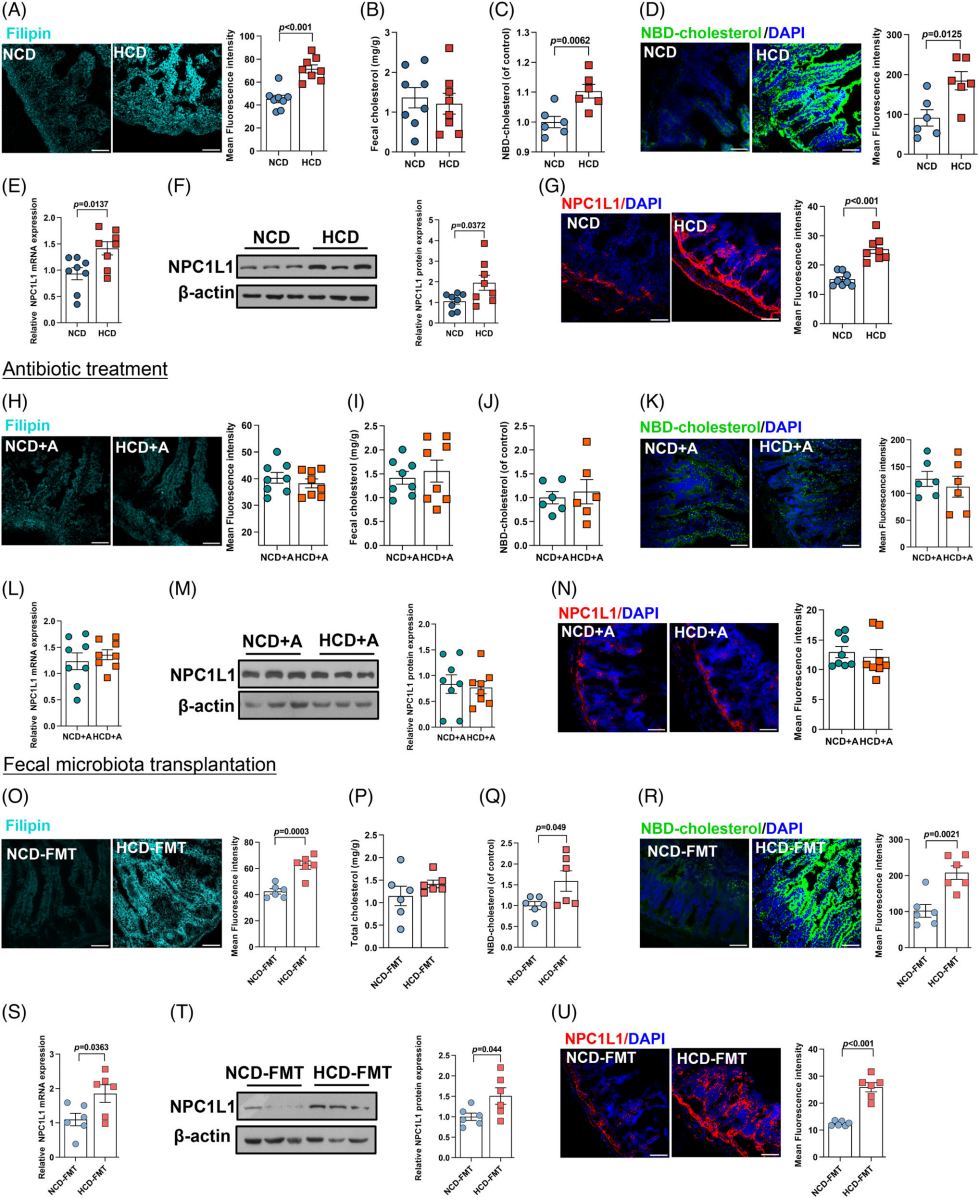
High chicken protein diet (HCD) upregulated intestinal cholesterol absorption in a gut microbiota‐dependent manner. (A, H, and O) Cholesterol accumulation determined by filipin staining in jejunum (scale bar represented 50 µm). (B, I, and P) Levels of total cholesterol in fecal samples. (C, D, J, K, Q, and R) Cholesterol absorption was determined by levels of NBD‐cholesterol in (C, J, and Q) circulation and (D, K, and R) jejunum. Expression of NPC1‐like intracellular cholesterol transporter 1 (*NPC1L1*) in jejunum at both (E, L, and S) mRNA and (F, M, and T) protein levels, and its (G, N, and U) density. Data were represented as mean ± SEM (*n* = 8 per group for A–N, and *n* = 6 per group for O–U). Statistical analysis was performed by unpaired *t* test.

To further delineate the mechanisms whereby HCD promoted atherosclerosis, we first determined the expression of genes involved in hepatic cholesterol metabolism and found no significant difference in mice fed with NCD or HCD (Figure S3A). We then interrogated intestinal cholesterol metabolism, which also played a crucial role in maintaining cholesterol homeostasis.^17^ Compared to mice fed with NCD, HCD administration resulted in a 1.5‐fold increase in cholesterol accumulation in the small intestine (Figure 3A). However, no obvious difference in the level of fecal cholesterol (Figure 3B) and genes involved in cholesterol flux and enterohepatic reabsorption of bile acids^17^ in the jejunum were observed (Figure S3B), suggesting that HCD exerted no effect on intestinal cholesterol excretion. On the other hand, intestinal cholesterol absorption was found to be markedly enhanced, as evidenced by an augmentation in 22‐(N‐7‐nitrobenz‐2‐oxa‐1,3‐diazo‐4‐yl)‐amino‐23,24‐bisnor‐5‐cholen‐3β‐ol) (NBD‐cholesterol) both in circulation (Figure 3C) and in the jejunum (Figure 3D). Consistently, the expression of NPC1‐like intracellular cholesterol transporter 1 (NPC1L1), a key transmembrane transporter responsible for intestinal cholesterol absorption,^17^ was 1.5‐fold higher in the jejunum of mice exposed to HCD, compared to those fed with NCD (Figure 3E). Moreover, both the protein expression (Figure 3F) and density (Figure 3G) of *NPC1L1* in the jejunum were also found to be remarkably higher in mice exposed to HCD. Similar to the observation in plaque formation, elimination of gut microbiota through sustained exposure to antibiotics cocktail abolished the discrepancies in intestinal cholesterol accumulation, absorption, and *NPC1L1* expression between NCD‐ or HCD‐fed mice (Figure 3H–N, Figure S3C,D). Moreover, mice colonized with microbiota from donors exposed to HCD demonstrated a substantially higher level of cholesterol accumulation and absorption in the jejunum compared to those receiving fecal microbial transplantation from mice fed with NCD (Figure 3O–R, Figure S3E,F). Moreover, jejunal *NPC1L1* expression was almost twofold higher in mice receiving fecal microbiota from mice fed with HCD (Figure 3S–U). Taken together, these findings implied that HCD promoted hypercholesterolemia depending on the presence of gut microbiota.

### 2.3 HCD modifies the composition and function of gut microbiota

**FIGURE 4:**
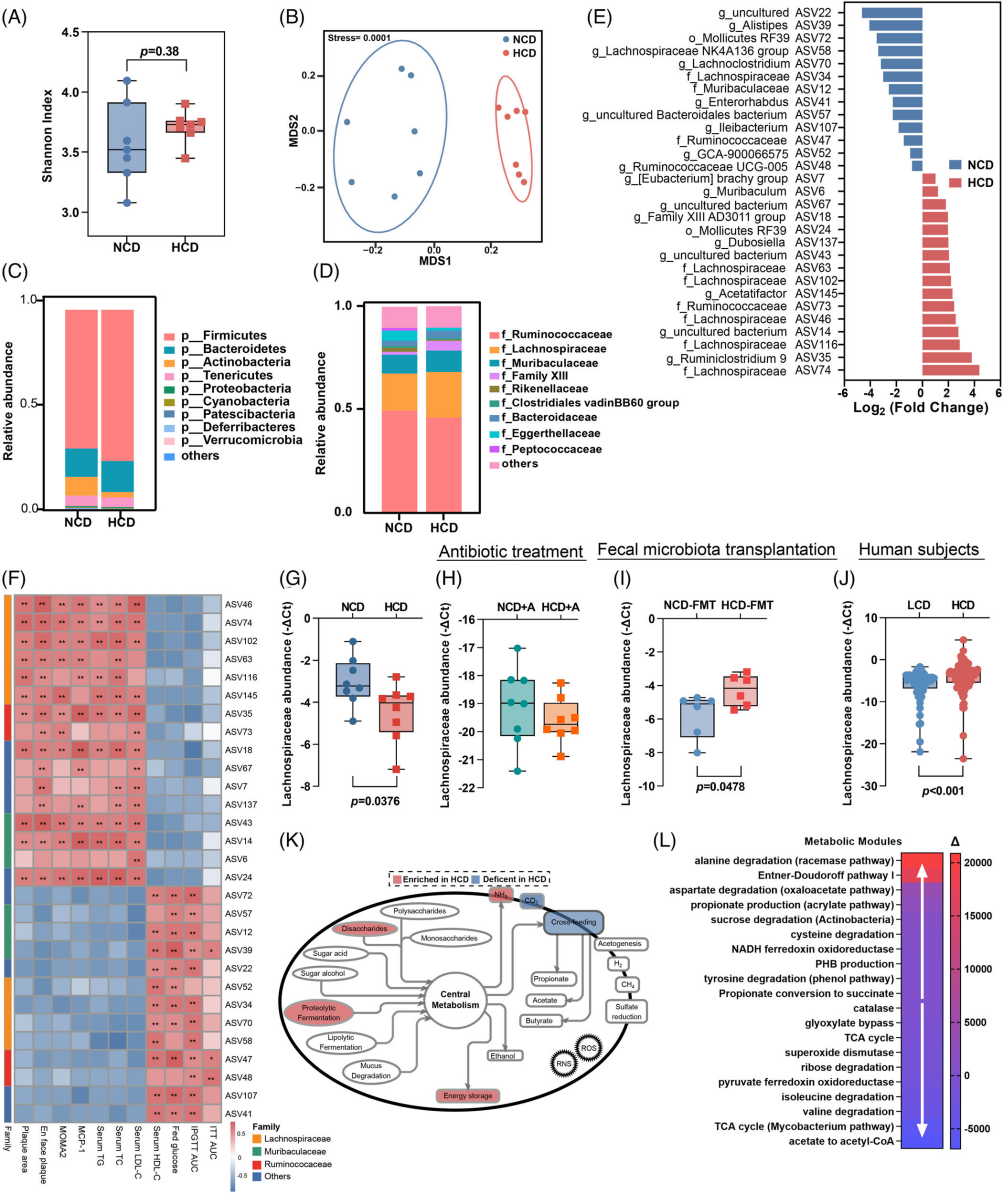
High chicken protein diet (HCD) disturbed the composition and function of gut microbiota. (A) Alpha‐diversity (Shannon index) of gut microbiota in mice fed with normal chicken protein diet (NCD) or HCD. (B) Multidimensional scaling with Bray–Curtis dissimilarity illustrating the separation of samples according to diet. Taxonomic summary of gut microbiota at (C) phylum and (D) family level. Each point represented one mouse. (E) Significantly altered amplicon sequence variants (ASVs). (F) Heatmap of the Spearman's correlations between significantly altered ASVs and phenotypes related to atherosclerosis. Red and blue indicated positive and negative correlations, respectively (*n* = 7 per group for A–F). Real‐time PCR was performed to determine the abundance of *Lachnospiraceae* family in mice (G) fed with NCD or HCD (*n* = 8 per group), (H) mice fed with NCD or HCD and exposed to long‐term antibiotics cocktail (*n* = 8 per group), (I) mice receiving fecal microbial transplantation from donors exposed to NCD or HCD (*n* = 6 per group), and (J) community residents with low or high intake of chicken protein (*n* = 69 per group). Data were represented as mean ± SEM. Statistical analysis was performed by unpaired *t* test. (K) Significantly altered metabolic processes and (L) modules in mice fed with HCD. Color scale indicated the mean difference in modules between HCD and NCD. An FDR adjusted *p* value < 0.1 was considered as significant. All *p* values were adjusted for multiple comparisons with Benjamini & Hochberg (BH) method.

To further elucidate how microbial metabolism mediates the atherogenic effect of HCD, an integrative analysis of 16S rDNA gene sequencing and plasma metabolomics were employed. Though there was no obvious difference in alpha diversity (Figure 4A), non‐metric multidimensional scaling ordination analysis based on amplicon sequence variants (ASVs) showed a significant separation of microbial composition in mice fed with NCD or HCD (Figure 4B). Specifically, compared to mice fed with NCD, *Fircumetes, Deferribacteres*, and *Patescibacteria* phylum (Figure 4C), and *Lachnospiraceae* and *family XIII* families (Figure 4D) were substantially enriched in mice exposed to HCD. A detailed compositional analysis further demonstrated that a total of 16 ASVs were increased, while 13 ASVs were decreased in the microbiota of mice fed with HCD (Figure 4E). Notably, over half the ASVs increased in mice fed with HCD belonged to the *Lachnospiraceae* family, a group of bacteria with proteolytic activity.^18^ Moreover, the majority of them (ASV63, ASV116, ASV145, ASV102, ASV74, and ASV46) demonstrated a strong correlation with plaque areas and unfavorable lipid profiles (Figure 4F). Using specific primers targeting the *Lachnospiraceae* family, we found that the increased abundance of *Lachnospiraceae* in mice fed HCD (Figure 4G) was diminished when exposed to long‐term antibiotics treatment (Figure 4H), whereas increased *Lachnospiraceae* abundance could be restored through microbiota transplantation from donors challenged with HCD (Figure 4I). Consistent with our findings in animal models, the abundance of *Lachnospiraceae* was also found to be considerably higher in subjects with a higher intake of chicken protein (Figure 4J), suggesting that *Lachnospiraceae* family functioned as the main bacteria mediating the pro‐atherogenic effect of HCD. Furthermore, functional interference demonstrated that proteolytic fermentation, ammonia production, and energy storage were notably augmented in mice fed with HCD (Figure 4K). More specifically, metabolic modules responsible for amino acid metabolism, such as alanine degradation, aspartate degradation, cysteine degradation, and tyrosine degradation, were preferentially enriched in mice exposed to HCD (Figure 4L). Altogether, the above findings suggested that expanded *Lachnospiraceae* and altered amino acid metabolism might be involved in the atherogenic effect of HCD.

### 2.4 3‐MH serves as the main microbial effector of HCD to enhanced atherosclerosis

**FIGURE 5:**
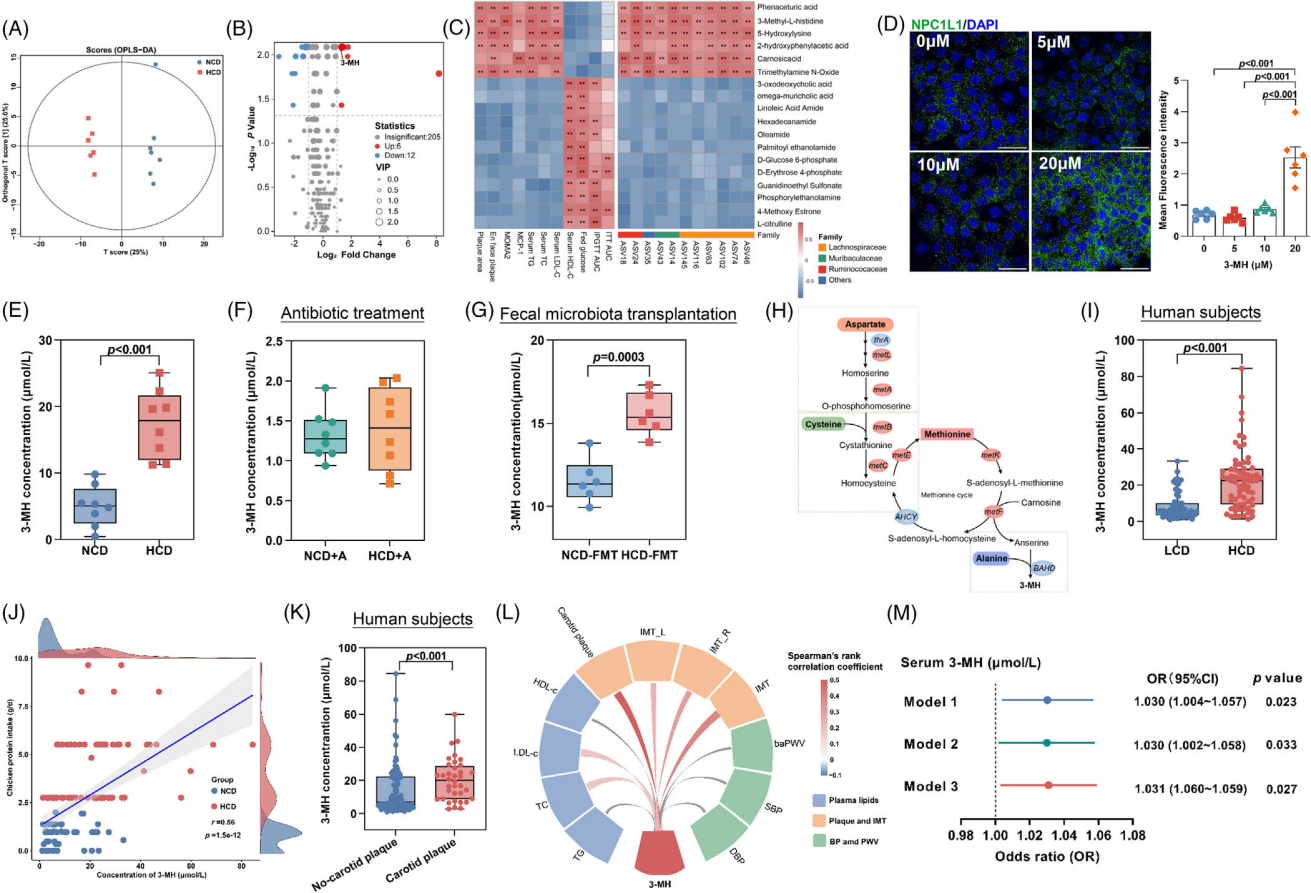
3‐Methyl‐L‐histidine (3‐MH) served as the main microbial effector of high chicken protein diet (HCD) to atherogenesis. (A) Score scatter plot of plasma metabolites in mice fed with normal chicken protein diet (NCD) or HCD. (B) Volcano plot analysis of metabolites significantly altered after 12‐week HCD treatment. Red and blue indicated increased and decreased metabolites, respectively, after HCD treatment. (C) Heatmap of Spearman's correlations between significantly altered amplicon sequence variants (ASVs), phenotypes related to atherosclerosis, and significantly altered metabolites after HCD intervention (*n* = 7 per group for A–C). (D) Density of NPC1‐like intracellular cholesterol transporter 1 (NPC1L1) in response to various dosages of 3‐MH (*n* = 6 independent experiments). Plasma levels of 3‐MH in mice (E) fed with NCD or HCD (*n* = 8 per group), (F) mice fed with NCD or HCD and exposed to antibiotics cocktail (*n* = 8 per group), and (G) mice receiving fecal microbial transplantation from donors fed with HCD or NCD (*n* = 6 per group). (H) Schematic summary showing the microbial production of 3‐MH. (I) Plasma levels of 3‐MH in subjects according to the intake of chicken protein (*n* = 69 per group). (J) Correlation between plasma 3‐MH levels and chicken protein intake derived from food questionnaire. (K) Plasma levels of 3‐MH in subjects according to the presence of carotid plaques. (L) Correlation between plasma 3‐MH and clinical parameters. (M) Crude and adjusted odds ratio for having carotid plaque with increasing levels of 3‐MH. Model 1: adjusted for none; Model 2: adjusted for BMI, diastolic blood pressure and total cholesterol; Model 3: Model 2 plus intake of red meat and plant protein. Statistical analysis was performed by one‐way analysis of variance followed by Tukey's test in (D), unpaired *t* test in (E–G), Wilcoxon signed‐rank test in (I), and Wilcoxon rank‐sum test in (K). An FDR adjusted *p* value < 0.1 was considered as significant in (C) and (L). All *p* values were adjusted for multiple comparisons with BH method.

Given that small molecules always functioned as transducers mediating the metabolic effect of gut microbiota,^15^ we next investigated how plasma metabolites linked HCD‐shaped microbiota to accelerated progression of atherosclerosis. Though *Lachnospiraceae* family was reported to be involved in the production of TMAO^16^ and SCFAs,^17^ these well‐known microbial metabolites showed no significant difference upon HCD or FMT challenge (Figure S4), suggesting that they were not involved in HCD‐induced atherosclerosis progression. However, orthogonal partial least squares‐discrimination analysis of metabolomics profiling showed a distinct metabolic profile between mice receiving NCD or HCD (Figure 5A), and volcano plot analysis further demonstrated that a total of six metabolites were significantly increased, while another 12 metabolites were substantially decreased after HCD administration (Figure 5B). Among them, 3‐MH, phenaceturic acid (PG), and 5‐hydroxylyysine (5‐HL) ranked as the top three metabolites enriched in mice exposed to HCD, and demonstrated a positive correlation with upregulated ASVs belonging to *Lachnospiraceae* family (ASV63, ASV116, ASV145, ASV102, ASV74, and ASV46), adverse cholesterol levels, and plaque areas (Figure 5C). To identify metabolites contributing to increased intestinal cholesterol absorption in mice challenged with an HCD, Caco‐2 cells were used to evaluate the effects of the top three metabolites on intestinal cholesterol metabolism. Incubation of 5‐HL or PG did not alter cholesterol absorption, or the expression of *NPC1L1* (Figure S5A–H), while 3‐MH was the only metabolite that induced a dose‐dependent increase in cholesterol absorption (Figure S5I,J) accompanied by a graded increase in the expression (Figure S5K,L) and density (Figure 5D) of *NPC1L1*.

Of note, the augmented levels of 3‐MH upon HCD challenge (Figure 5E) were almost completely abolished in mice exposed to antibiotics cocktail (Figure 5F), but reversed to a level comparable to those observed in mice fed with HCD by receiving fecal microbiota from donors challenged with HCD (Figure 5G). More importantly, such an increase in 3‐MH production was consistent with the functional interference of gut microbiota. Enhanced degradation of alanine, aspartate, and cysteine upon HCD challenge led to an increase in methionine metabolism, which ultimately resulted in an increased production of 3‐MH (Figure 5H, Figure S6A). The increase in the abundance of intermediate metabolites and precursors along the pathways responsible for 3‐MH production was absent in mice exposed to antibiotics cocktail (Figure S6B), but reversed in mice receiving FMT from donors fed with HCD (Figure S6C), indicating that 3‐MH was a microbiota‐related metabolite. Furthermore, in line with the observation in mice, the plasma level of 3‐MH in individuals with high intake of chicken protein was fourfold of those with a low consumption (Figure 5I), and showed a close correlation with the intake of chicken protein calculated from food questionnaires (Figure 5J). Intriguingly, such an increased level of circulating 3‐MH was also found in subjects with carotid plaques compared to those without (Figure 5K), and demonstrated a positive association with carotid intima‐media thickness and atherogenic cholesterols (Figure 5L). After correcting for major confounders, increased 3‐MH remained an independent risk factor for sub‐clinical atherosclerosis (Figure 5M). Taken together, findings from both mouse model and human studies demonstrated that 3‐MH might be the microbial effector of HCD to its pro‐atherogenic effect.

### 2.5 3‐MH aggravates atherosclerotic plaque formation via enhancing NPC1L1‐mediated intestinal cholesterol absorption

**FIGURE 6:**
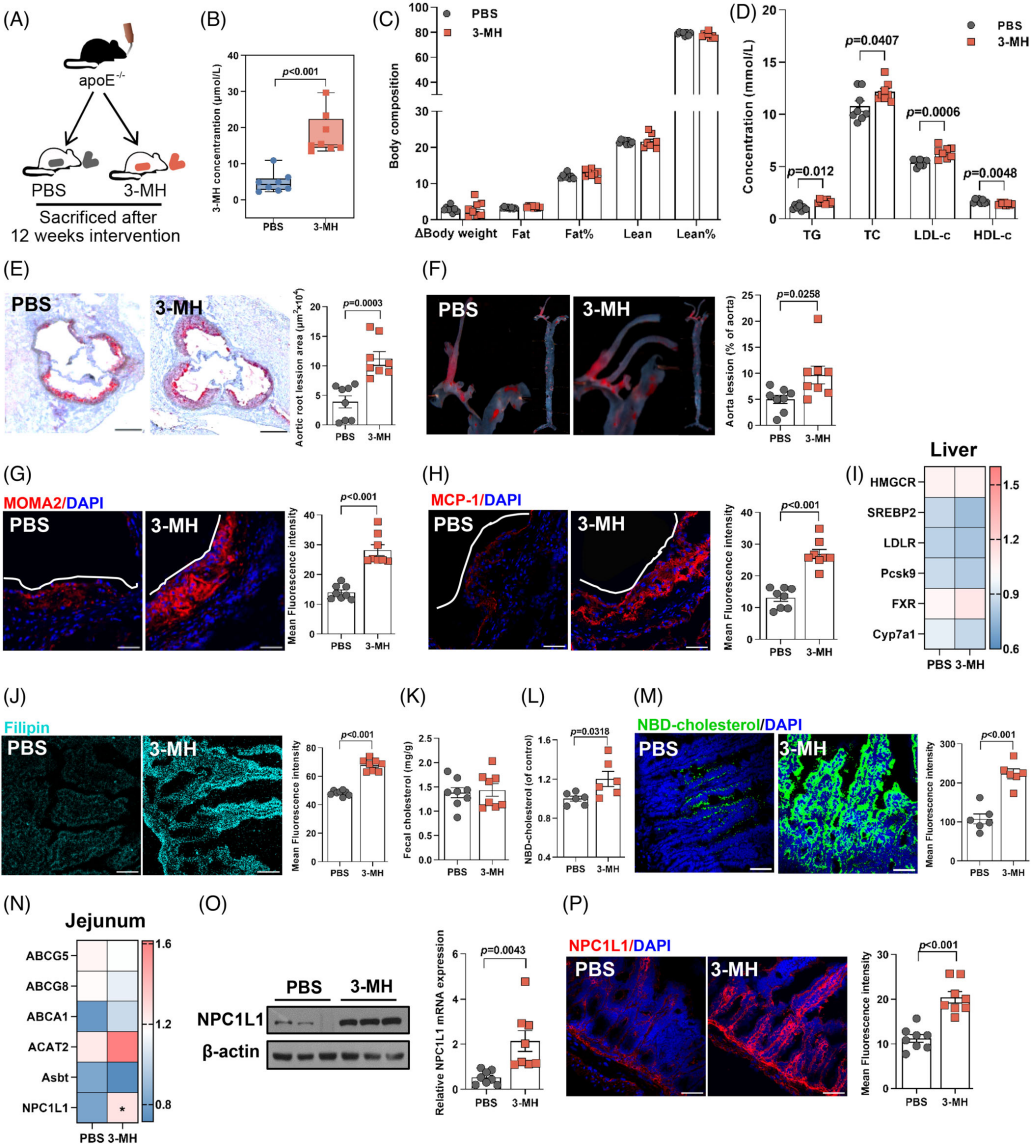
3‐Methyl‐L‐histidine (3‐MH) enhanced intestinal cholesterol absorption and promoted the progression of atherosclerosis. (A) Experimental design. (B) Plasma levels of 3‐MH. (C) Body composition. (D) Serum levels of triglyceride, total cholesterol, high‐density lipoprotein cholesterol, and low‐density lipoprotein cholesterol. (E) Cross‐sections of aortic sinuses stained with oil red O (scale bar represented 200 µm). (F) En face staining of the entire aorta (scale bar represented 20 mm). Macrophage infiltration and inflammation activation in aortic sinus determined by immunostaining for (G) *MOMA2* and (H) *MCP‐1*, respectively (scale bar represents 50 µm). (I) Expression of genes involved in hepatic cholesterol biosynthesis and transport. (J) Cholesterol accumulation determined by filipin staining in jejunum (scale bar represented 50 µm). (K) Levels of total cholesterol in fecal sample. Cholesterol absorption was determined by levels of NBD‐cholesterol in (L) serum and (M) jejunum. (N–P) Expression of NPC1‐like intracellular cholesterol transporter 1 (*NPC1L1*) in jejunum at both (N) mRNA and (O) protein levels, as well as (P) its density. Data represented as mean ± SEM (*n* = 8 per group). Statistical analysis was performed by unpaired *t* test.

To further interrogate whether 3‐MH mimicked the pro‐atherogenic effect of HCD, mice were gavaged with 3‐MH or sterile phosphate buffered saline (PBS) for 12 weeks (Figure 6A, *n* = 8 per group). Daily gavage of 3‐MH led to over four−fold increase in circulating 3‐MH (Figure 6B), resulting in a level comparable to that observed in mice fed with HCD (Figure 5E) and individuals with high intake of chicken protein (Figure 5I). Exposure to 3‐MH exerted no obvious effect on food intake (Figure S7A), body composition (Figure 6C), and glycemic control (Figure S7B–D), but led to a remarkable increase in atherogenic cholesterols (Figure 6D). Compared to mice gavaged with PBS, 3‐MH resulted in a 200% increase in plaque areas (Figure 6E,F), accompanied by an augmentation in macrophage infiltration and MCP‐1 expression (Figure 6G,H). Similar to the effect of HCD, 3‐MH had no obvious effect on the gene expression responsible for hepatic cholesterol metabolism (Figure 6I), but resulted in a 1.4‐fold increase in intestinal cholesterol accumulation (Figure 6J), with no obvious effect on cholesterol excretion (Figure 6K). As expected, 3‐MH treatment also led to a marked increase in cholesterol in the circulation (Figure 6L), and an enhanced intestinal cholesterol absorption (Figure 6M) through the augmentation of NPC1L1 expression and density (Figure 6N–P). Moreover, inhibition of NPC1L1 through either specific inhibitor (Figure S8A,B) or siRNA silencing (Figure S8E,F) largely abolished the activation of intestinal cholesterol absorption and accumulation induced by 3‐MH (Figure S8C,D,G,H). Collectively, the above data supported a regulatory circuit in which elevated microbiota‐derived 3‐MH induced by HCD upregulated intestinal cholesterol absorption via NPC1L1, which subsequently promoted the progression of atherosclerosis.

### 2.6 HNF1A is required for NPC1L1‐mediated intestinal cholesterol absorption by 3‐MH

**FIGURE 7:**
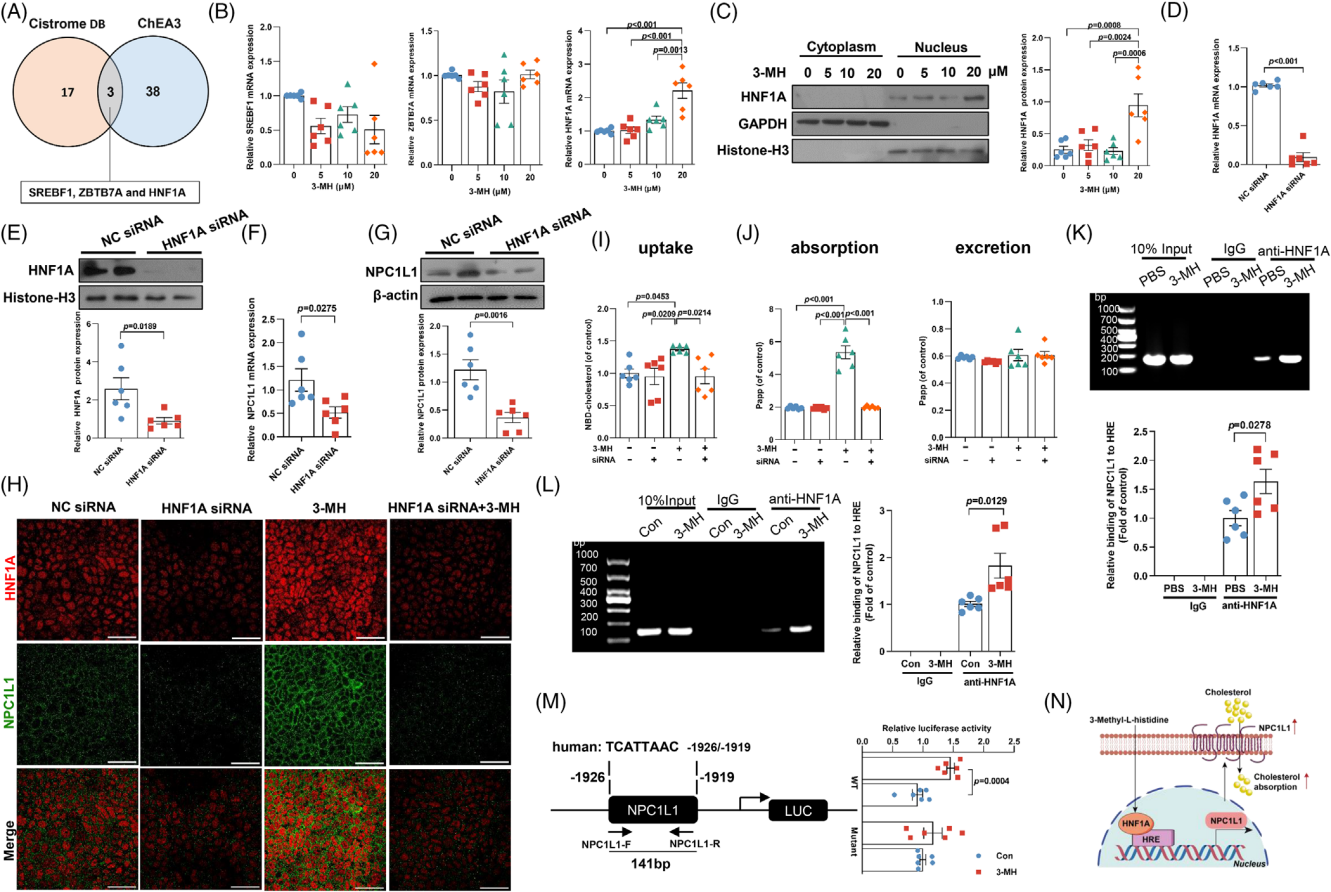
3‐Methyl‐L‐histidine (3‐MH) promoted cholesterol absorption via activating *HNF1A‐NPC1L1* axis. (A) Three potential regulators of NPC1‐like intracellular cholesterol transporter 1 (*NPC1L1*) transcription derived from the intersection of Cistrome DB Toolkit and ChEA3 database. (B) Expression of potential transcription factors upon 3‐MH challenge. (C) Expression of *HNF1A* in nucleus and cytoplasm upon 3‐MH challenge. (D–H) Caco‐2 cells were transfected with *HNF1A*‐specific siRNA for 48 h before analysis. *HNF1A* expression at (D) mRNA level and (E) protein level in the nucleus. *NPC1L1* expression at (F) mRNA and (G) protein levels and its (H) density. (I) Cholesterol accumulation and (J) cholesterol absorption and secretion in Caco‐2 cells. (K) Jejunum from mice fed with PBS or 3‐MH for 12 weeks, and (L) cells incubated with 3‐MH or PBS were analyzed for *HNF1A* binding to *NPC1L1* promoter by using the chromosome‐immunoprecipitation assay. (M) Cells were transfected with empty vector or the pGL3‐NPC1L1 promoter plasmid with or without a mutated *HNF1A*‐binding site and then treated with 3‐MH (20 µM) for 12 h before measuring *NPC1L1* promoter luciferase activity. Data were represented as mean ± SEM (*n* = 6 independent experiments for B‐I and L‐M, and *n* = 6 per group for K). Statistical analysis was performed by unpaired *t* test. (N) Schematic summary illustrating the potential mechanism whereby high chicken protein diet (HCD) accelerated the progression of atherosclerosis.

To further understand how 3‐MH induced NPC1L1 activation, we first performed in silico analysis to screen for putative transcription factors most likely to regulate NPC1L1. Based on the intersection of Cistrome DB toolkit^19^ (Figure S9A) and ChEA3 database^20^ (Figure S9B), sterol regulatory element binding transcription factor 1, zinc finger and BTB domain containing 7A, and hepatic nuclear factor 1A (HNF1A) ranked as the top three potential regulators mediating the transcription of intestinal NPC1L1 (Figure 7A). Then, Caco‐2 cells were exposed to 3‐MH at the concentration detected in subjects with low or high intake of chicken protein diet. Of the three potential regulators, only *HNF1A* exhibited a marked increase upon 3‐MH exposure at both mRNA and protein level in the nucleus (Figure 7B,C), indicating that *HNF1A* served as the potential regulator responsible for the activation of NPC1L1‐mediated cholesterol absorption by 3‐MH. Notably, 3‐MH‐induced *NPC1L1* activation and membrane enrichment were almost completely abolished after pretreatment with specific siRNA targeting *HNF1A* (Figure 7D–H). Consistently, 3‐MH‐induced cholesterol accumulation and absorption were reduced by 1.4‐fold and 2.7‐fold, respectively, while no effect on cholesterol excretion was found after *HNF1A* silencing (Figure 7I,J). Moreover, chromatin immunoprecipitation analysis showed that incubation of 3‐MH significantly enhanced the binding ability of HNF1A to NPC1L1 promoter region in both mice challenged with 3‐MH (Figure 7K) and Caco‐2 cells exposed to 3‐MH incubation (Figure 7L). To directly test the role of the *HNF1A*‐binding site of *NPC1L1* promoter in mediating the 3‐MH effect, we further performed site‐directed mutagenesis: mutation of the putative *HNF1A*‐binding site at nucleotide −1919/−1926 (Mut) relative to the transcription start site substantially attenuated 3‐MH‐induced *NPC1L1* promoter transcription activity (Figure 7M), suggesting that *HNF1A* activation was necessary for 3‐MH‐induced *NPC1L1* transcription. In summary, the above findings indicated that *HNF1A‐NPC1L1* signaling axis was required for 3‐MH‐induced intestinal cholesterol absorption (Figure 7N).

## 3 DISCUSSION

The benefits of high chicken diet on cardiovascular health have long been controversial. Here, we provided evidence in both humans and animal models that high intake of chicken protein accelerated the progression of atherosclerosis through an increased production of 3‐MH, resulting from an expansion of *Lachnospiraceae* family and enhanced methionine degradation in the microbiome. Mechanically, increased 3‐MH facilitated atherosclerosis progression through NPC1L1‐mediated intestinal cholesterol absorption in a *HNF1A*‐dependent manner (Figure 7N).

High protein diet has been popularized as a strategy for weight loss since 1960s mainly due to its dual benefits of improving satiety and decreasing fat mass, which was always accompanied by an improvement in glycemic control.^21^ Among various sources of protein, white meat, represented by poultry such as chicken, was considered to be healthier due to its lower amount of saturated fatty acids, higher proportion of polyunsaturated fatty acids than red meat^22^ and also a more balanced pattern of essential amino‐acids than plant protein.^23^ Consistent with the remarkable effect on glucose metabolism and body weight loss reported in different ethnics,^24^ we did find an obvious reduction in fat mass and enhancement in glucose disposal (Figure 2B, Figure S2B–D). However, almost all these studies, either in the form of observational cohorts or randomized controlled trials, were conducted in subjects with type 2 diabetes or metabolic syndrome, and few of them lasted for an enough long time to observe the cardiovascular effect. Here, using a diet mimic the popular formula advocated by the lay media, we showed that despite a positive effect on glucose control, long‐term adherence to HCD promoted hypercholesterolemia and facilitated the progression of atherosclerosis (Figure 2C–E), lending further support to the notion that optimal glycemic control cannot guarantee lower risk for cardiovascular disease. Such an observation was not unusual considering the observation that the association between HbA1c and cardiovascular mortality was J‐shaped in patients with type 2 diabetes.^25^ Similar to the findings in prospective cohorts, intensive glycemic control did not result in a significant reduction in cardiovascular risk in several large‐scale clinical trials, such as the Action to Control Cardiovascular Risk in Diabetes trial involving 10,251 individuals^26^ and the multi‐center Veterans Affairs Diabetes Trial.^27^ Additionally, though did not differentiate the source of protein, elevation of dietary protein to the 40 kcal% was also reported to accelerate atherosclerosis progression via activating mammalian target of rapamycin signaling in mice.^15^ Collectively, the above findings highlight the importance of incorporation of vascular health into consideration when designing diet for body weight management and argued against its long‐term application.

Dietary factors are among the most potent modulators of microbiota composition and function, which has been proved to be an essential organ responsible for the various dimensions of host physiology.^28^ Therefore, it would be pivotal to determine whether gut microbiota was directly involved in atherogenic effect of HCD and, if so, what was the underling mechanism. 16s rDNA sequencing demonstrated that long‐term HCD led to a substantial increase in the relative abundances of *Lachnospiraceae* family and methionine degradation. Interestingly, enrichment of *Lachnospiraceae* and *Ruminococcaceae* had also been reported to be associated with an enhanced production of TMAO in humans. Additionally, the harmful effect of high‐fat diet on metabolism was found to be associated with an increase in indole and its derivatives resulting from increased level of *Lachnospiraceae* and *Muribaculaceae*.^29^ Collectively, increased abundance of *Lachnospiraceae* family may function as a common link between various diets and their deteriorative effect on host metabolism. Moreover, integrative analysis with targeted metabolomics further demonstrated that increased production of microbiota‐derived 3‐MH, resulting from enhanced methionine degradation, served as the key microbial effector mediating the pro‐atherogenic effect of HCD. Previously reported to be a biomarker for elevated muscle degradation, the normal concentration of 3‐MH was less than 5.9 µM in the circulation.^30^ However, HCD led to approximately three‐ and sixfold increase in the levels of 3‐MH in the plasma (Figure 5E), which was abolished when exposed to antibiotics treatment (Figure 5F) but restored by fecal microbial transplantation (Figure 5G), suggesting that its production was highly dependent on the presence of gut microbiota. Therefore, though 3‐MH can either be obtained from diet or produced by host and gut microbiota,^31^, ^32^ we proposed that difference in circulating 3‐MH induced by HCD was mainly due to microbial metabolism under our experimental conditions. Furthermore, consistent with the pro‐atherogenic effect of 3‐MH observed in our study, a recent metabolomics analysis in patients with heart failure showed that higher 3‐MH was associated with worsening of heart failure and all‐cause mortality.^33^ In a similar fashion, dietary phenylalanine, which occurs naturally in many protein‐rich foods, such as milk, eggs, and meat, was transformed into phenylacetyl glutamine by gut microbiota and contributed to the development of cardiac disease.^34^ Collectively, these findings reveal that microbial metabolism of dietary protein may serve as an attractive preventive target for cardiovascular diseases. Prebiotics or prebiotics, which may inhibit the production of microbial 3‐MH, may serve as a dietary strategy to ensure the cardio‐metabolic health in individuals with a high intake of chicken protein.

Cholesterol homeostasis has long been proved to be an essential lever to cardiovascular health, which is achieved through the intricate interplay of intestinal cholesterol absorption, plasma lipoprotein uptake, de novo biosynthesis, and cholesterol catabolism and excretion. Mounting evidence have demonstrated a notable modulatory effect of various dietary interventions on the various processes of cholesterol metabolism. On the one hand, diet rich in fiber and phytosterols were able to decrease LDL‐c by inhibiting intestinal cholesterol absorption.^35^ Likewise, phytochemicals, such as curcumin and anthocyanin, demonstrated a strong hypolipidemic effect mainly though suppressing hepatic cholesterol biosynthesis and enhancing reverse cholesterol transport.^36^ Considering that there is still a large portion of individuals fail to reach the target cholesterol levels recommended by guidelines despite a wide prescription of statin and ezetimibe,^37^ it is important to take full advantage of the synergistic effect of dietary intervention for the management of cholesterol homeostasis. Through the combination of in vitro and in vivo experiments, we showed that increased 3‐MH promoted the binding ability of *HNF1A* to *NPC1L1* promoter, which in turn enhanced intestinal cholesterol absorption and plaque formation. As an important transcription factor regulating a number of genes involved in lipid and metabolism, variants of the gene encoding HNF1A had been reported to be associated with several cardiovascular risk factors.^38^ Similar to our observation, lycopene was also reported to delay the progression of atherosclerosis through downregulating *HNF1A‐NPC1L1* signaling.^39^ Similarly, transforming growth factor β induced factor homeobox 1 activation, a transcriptional repressor, was reported to alleviate intestinal cholesterol absorption via suppressing *NPC1L1*‐mediated cholesterol uptake in a *HNF1A*‐dependent manner.^40^ Taken together, these findings suggest that *HNF1A‐NPC1L1* axis may serve as a common regulator and potential therapeutic target to modulate cardiovascular health, especially in individuals with an over‐consumption of chicken protein.

There also exist some limitations that need to be acknowledged to provide a comprehensive interpretation of our findings. Associations of habitual chicken protein intake and 3‐MH with sub‐clinical atherosclerosis were analyzed in cross‐sectional study from a single center. Prospective cohorts from various ethnics and randomized controlled trials are warranted to further validate this association. Moreover, though our results have demonstrated a robust underlying biological association between HCD‐derived 3‐MH and accelerated atherosclerotic plaque formation in apoE^−/−^ mice, a well‐established model for atherosclerosis,^40^, ^41^ future endeavors incorporating more animal models that can capture the pathological process of atherosclerosis in humans are necessary.

## 4 CONCLUSION

In summary, our findings uncover increased microbial production of 3‐MH as the main molecular effector linking the deteriorating effect of HCD on vascular health via the activation of *HNF1A‐NPC1L1* axis and also highlight the importance of taking microbial metabolism into consideration when designing diet regimes for the management of cardiovascular health.

## 5 MATERIALS AND METHODS

### 5.1 Study population

Subjects with carotid ultrasonography, food frequency questionnaire, and plasma samples were recruited from the Community Healthcare Center of Chashan Town (Dongguan, China) through flyers and advertisement to explore the association of chicken protein intake and probability of carotid atherosclerosis. At least 57 individuals per group were needed, based on the formula for paired case–control study, with a power of 90%, 5% significance, and an estimated effect size of 3.79.^41^, ^42^, ^43^ The study was approved by the Ethics Committee of School of Public Health, Sun Yat‐Sen University (L2017‐001). Written informed consents from all participants were obtained and the detailed inclusion and exclusion criteria for this study were as follows: *Inclusion criteria*: (1) local residents aged between 35 and 75 years; (2) no severe disability, cancer or malignant tumors; (3) absence of any infections within previous month; and (4) absence of any antibiotics within 3 months before sample collection. *Exclusion criteria*: (1) missing information for food frequency questionnaire; (2) missing plasma samples; (3) implausible energy intake levels (<500 or >3500 kcal/day); (4) regular consumption (≥3 times per week) of probiotics or prebiotics within 1 month before participation; (5) acute illness or current evidence of acute or chronic inflammatory disease, established cardiovascular diseases, such as coronary disease, stroke, and heart failure; and (6) other metabolic diseases that may affect gut microbiota, including type 1 or type 2 diabetes mellitus, biliary obstructive diseases, acute or chronic cholecystitis, acute or chronic viral hepatitis, cirrhosis, diarrhea, hyperthyroidism or hypothyroidism, chronic renal insufficiency, cancer, pregnancy, and gastrointestinal diseases.

Finally, a total of 138 subjects were eligible for analysis. The intake of various nutrients was calculated according to the Dietary Composition Database of Chinese. The median intake of chicken protein in the entire South China Cohort was 2.5 g/day (data not shown). Subsequently, a total of 69 subjects with a high intake of chicken protein (defined as higher than the median intake of this population), and another 69 individuals with a low intake of chicken protein (defined as lower than median intake of this population) matched for age and sex, were included in this study. More details about data collection and biological processing were provided in Supporting Information.

### 5.2 Animal studies

All animal experiments were approved by the Animal Care and Utilization Committee of Sun Yat‐sen University (No. 2018‐009). Eight‐week apoE^−/−^ mice in C57BL/6J background were obtained from Charles River Laboratories. All mice were housed in colony with a 12‐h‐light/12‐h‐dark cycle in a temperature‐controlled environment.

#### 5.2.1 Experiment 1: mice fed with normal or high chicken protein diet

Male apoE^−/−^ mice were fed with either NCD (in terms of energy, 15% from chicken protein, Medicience Ltd., China), or HCD (in terms of energy, 30% from chicken protein, Medicience Ltd., Jiangsu, China) for 12 weeks to mimic normal and over‐consumption of chicken protein in humans (Table S1). The chicken protein used for both diets was obtained from BIN BarnDad's 100% CHXN Chicken Protein Isolate (Cranberry). All diets were autoclaved with irradiation before use.

#### 5.2.2 Experiment 2: long‐term antibiotics cocktail treatment

To explore the role of gut microbiota in the pro‐atherogenic effect of HCD, antibiotics cocktail consisting of vancomycin (0.5 g/L), ampicillin (1 g/L), neomycin (1 g/L), and metronidazole (1 g/L) were given in autoclaved drinking to mice fed with NCD or HCD throughout the intervention period (12 weeks) and were replenished every 3 days, as described.^44^

#### 5.2.3 Experiment 3: fecal microbial transplantation

To further confirm the role of microbial metabolism in the pro‐atherogenic effect of HCD, fecal microbial transplantation (FMT) was performed as previously described.^44^ Briefly, fresh feces from apoE^−/−^ mice fed with NCD or HCD for 12 weeks (donor mice) were collected, weighed, and homogenized in pre‐reduced 0.5% cysteine/PBS followed by filtration through a 100 µm strainer in an anaerobic chamber. Fecal slurries were aliquoted and stored at −80°C until use. Recipient apoE^−/−^ mice were administered with antibiotics cocktail for 1 week to eliminate their gut microbiota, followed by a 3‐day wash‐out period. Then, fecal slurries were orally administered three times per week for a total of 12 weeks in recipient mice fed with normal chow diet (D10012M, Research Diets, Inc.). Mice received fecal slurry from NCD‐fed donors were designated as NCD‐FMT group, while mice received fecal slurry from HCD‐fed mice were designated as HCD‐FMT group.

#### 5.2.4 Experiment 4: 3‐MH treatment

To further interrogate the effect of 3‐MH, apoE^−/−^ mice fed with NCD were administrated with sterile PBS or 3‐MH (20 mg/kg per day) for a total of 12 weeks. Diet intake and body weight were recorded weekly and body composition were measured with an NMR system (Niumag) as previously described.^45^

### 5.3 Caco‐2 culture and treatment

The human colorectal adenocarcinoma cell line Caco‐2 was obtained from ATCC and cultured in modified Eagle medium (MEM) containing 20% fetal bovine serum, 1% non‐essential amino acid, and 1% penicillin‐streptomycin (Gibco, Thermo Fisher Scientific). The cells were incubated at 37°C in a humidified atmosphere of 5% CO_2_. When reaching 80% confluence, the cells were treated with various concentrations of 5‐HL (0, 0.2, and 0.7 µmol/L), PG (0, 15, and 50 µmol/L), or 3‐MH (0, 5, 10, and 20 µmol/L) for 12 h prior to analysis.

More experimental details were provided in Supporting Information.

### 5.4 Statistical analyses

Continuous data were subjected to the Kolmogorov–Smirnov test and Shapiro–Wilk test to determine normality and were expressed as the mean ± SEM deviation unless otherwise stated. Non‐parametric Wilcoxon rank‐sum test, unpaired *t*‐test or one‐way analysis of variance followed by Tukey's test was used as appropriate. Conditional logistic regression was employed to estimate the odds ratio (OR) and 95% confidence interval (95% CI) of dietary chicken protein intake or plasma 3‐MH and risk for sub‐clinical atherosclerosis. Multivariable conditional logistic regression analyses were adjusted for body mass index (BMI), diastolic blood pressure and total cholesterol, as well as intakes of red meat and plant protein. All statistical analyses were performed using GraphPad Prism 9 (Graphpad Software) or R software, version 4.0.3 (R Core Team, https://www.R‐project.org/).

## AUTHOR CONTRIBUTIONS

Y.L. designed the study and interpreted the results. S.S.Z. performed the majority of the animal experiments and data analyses. L.D.L, B.Q.Y., and J.L.H. helped with 16s rDNA analysis and metabolomics analysis. Y.W.Z. and Y.X.X. helped with the animal studies. J.Y.Z. and W.K.L. helped with the recruitment of study participants. S.S.Z., Y.L., and M.X. wrote and edited the manuscript. All authors have read and approved the final manuscript.

## CONFLICT OF INTEREST STATEMENT

The authors declare no conflicts of interest.

## ETHICS STATEMENT

The human study was approved by the Ethics Committee of School of Public Health, Sun Yat‐Sen University (L2017‐001). All animal experiments were approved by the Animal Care and Utilization Committee of Sun Yat‐sen University (No. 2018‐009).

## Supporting information



Supporting information

## Data Availability

Multi‐Omics data from this study have been deposited in publicly accessible databases. The 16S rDNA sequencing data have been deposited at the NCBI Sequence Read Archive under BioProject: PRJNA887908. The metabolomics data can be accessed at EBI‐MetaboLights under the BioProject: MTBLS12010.
