# Supplementary material for: Microbiota‐derived 3‐Methyl‐L‐histidine mediates the proatherogenic effect of high chicken protein diet
Source: MedComm (2020). 2025 Feb 13;6(2):e70090. doi: 10.1002/mco2.70090 (PMC11822454; doi:10.1002/mco2.70090)
Supplement: Supplementary file 1 — Supporting information [file MCO2-6-e70090-s001.pdf]

Supporting information

Microbiota-derived 3-Methyl-L-histidine mediates the proatherogenic effect of high chicken protein diet

Shanshan Zhu[1], Ludi Liu[2], Yawen Zhao[1], Bingqi Ye[2], Jialin He[1], Wenkang Li[1], Yingxi Xu[2], Jiangyuan Zhu[1], Min Xia[1], Yan Liu[1, *]

[1]Guangdong Provincial Key Laboratory of Food, Nutrition and Health, and Department of Nutrition, School of Public Health, Sun Yat-sen University, Guangzhou P.R. China
[2]Guangdong Provincial Key Laboratory of Food, Nutrition and Health, and Department of Statistics and Epidemiology, School of Public Health, Sun Yat-sen University, Guangzhou, P.R. China

[*]**Correspondence:** Yan Liu, Department of Nutrition, School of Public Health, Sun Yat-sen University; E-mail: liuyan215@mail.sysu.edu.cn.

**Contents**	**Pages**
Supplementary Methods	2-10
Supplementary references	11
Figure S1.	12
Figure S2.	13
Figure S3.	14
Figure S4.	15-16
Figure S5.	17-18
Figure S6.	19-20
Figure S7.	21
Figure S8.	22
Figure S9.	23
Table S1. Macronutrient composition and calories of NCD and HCD.	24
Table S2. Primer sequences for qRT-PCR	25

Supplementary Methods

Collection of biological samples and covariates

Body weight, height, systolic blood pressure and diastolic blood pressure were measured by trained staffs. Blood pressure was measured on the right upper arm in the sitting position after at least 10-15 min of rest using a validated digital automatic analyzer (Omron HEM-7136). A detailed questionnaire including demographic characteristics, medical history, current medication and lifestyles was performed by trained staffs in a face-to-face manner. Dietary intake was collected by 3-day food record (2 separate weekdays and 1 weekend), and analyzed by NutriStar software (Zhending Inc, Shanghai, China).

The brachial-ankle pulse wave velocity (baPWV) and ankle-brachial index (ABI) were measured simultaneously with an automatic waveform analyzer (BP-203 PRE III, Omron Health Medical, Dalian, China). Since the measurement of baPWV measurements may be biased in patients with severe atherosclerosis in lower legs, when bilateral ABI were less than 0.9 or substantial side differences (more than 1000 cm/s) occurred, the subjects were excluded from the analysis. When unilateral ABI was less than 0.9, we only considered the baPWV of the other side as the final baPWV reading, otherwise, the average value of the left- and right-side baPWV was used as the final reading. Carotid ultrasound was performed by experienced technologist (Aplio400, TOSHIBA, Japan). Ultrasound parameters were preset with the use of a special intima-media thickness (IMT) program that kept constant the postprocessing map, dynamic range, persistence, frame rate, and power output, and transmit gain control was altered to obtain optimal quality images. Measurements were made of both right and left carotid arteries. An artery was classified as being affected by plague if there was a localized thickening>1.2 mm.

Blood samples were collected between 8:00 to 9:00 after an overnight fast for 10-12h. Samples were processed immediately and all biochemical measurements were performed within 3 hours in the central laboratory of community healthcare center after collection via an automated analyzer (Mindray BS600, Mindray, Shenzhen, China).

Participants received MGIEasy stool collection kit containing a room temperature stabilizing reagent that preserves metagenomic samples during study visit (the same day with blood sample collection). Samples were stored at -20°C at the study visit location for a maximum of 1 day before transportation to the central freezer at -80°C

until analysis. Stool DNA was extracted from frozen human stools using MagMAXTM Microbiome Ultra Nucleic Acid Isolation kit (Thermo Fisher Scientific, MA, USA).

Glucose and Insulin Tolerance Tests in mice

For glucose tolerance test, $apoE^{-/-}$ mice were fasted for 14h overnight. Glucose (1.5 g/kg body weight) was injected intraperitoneally and blood glucose level was tested from tail vein at 0, 15, 30, 60, 90 and 120 min with a handheld glucometer (GA-3, Sinocare Inc., Hunan, China). For insulin tolerance test, $apoE^{-/-}$ mice were fasted for 4h. Insulin (0.75U/kg body weight) was injected intraperitoneally and blood glucose level was tested from tail vein at 0, 15, 30, 60, 90 and 120 min with a handheld glucometer.

Determination of circulating lipids in mice

TG, TC, LDL-c and HDL-c in the circulation were measured using an automated biochemical analyzer (Mindray Global. Inc) with TG, TC, LDL-c and HDL-c assay kit (Mindray Global. Inc, Shenzhen, China).

Cholesterol absorption in mice

Mice fasted overnight were orally gavaged with 0.4 mg NBD-cholesterol (Thermo Fisher Scientific, USA) in 200 µL corn oil. After 3 hours, the mice were euthanized and the intestine were harvested and subjected to analysis as previously described[1]. Jejunum was excised into pieces and embedded in tissue-tek OCT and stored at -80°C until analysis. The tissue samples were sectioned (4 µm) with a cryostat, fixed with 4% paraformaldehyde, and detected at 488 nm with a fluorescence microscope. The NBD-cholesterol in the serum was extracted by hexane-water (1:1, vol/vol) and dissolved with 10 mM Taurocholic acid sodium salt for fluorescence measurement at an excitation of 485 nm and emission of 535 nm.

Quantitative analysis of atherosclerotic lesions in mice

After euthanasia, the entire length of aorta with the heart attached were harvested and the upper half of the heart with aortic root was embedded in OCT compound (Sakura Finetek Japan, Tokyo, Japan) and cryosectioned to 8 µm. The cross-sectional areas were stained with Oil Red O (Sigma-Aldrich, Darmstadt, Germany) to detect

lesions and counterstained with hematoxylin (Servicebio, Beijing, China). After removal of adventitial adipose tissue, the remainder of the aorta were cut open longitudinally and stained with Oil Red O. The quantification of lesion area and size was performed with Image J software (National Institutes of Health, Baltimore, MD).

Immunofluorescence staining and quantification

Aortic roots embedded in tissue-tek OCT (Sakura Finetek Japan, Japan) were cut into 8 μm sections, followed by blocking with goat serum for 10 min at room temperature. Slides were subsequently incubated with primary antibodies (MOMA2: Abcam, ab8304, 1:400; MCP-1: Thermo Fisher Scientific, MA5-17040, 1:400; NPC1L1: Santa Cruz, Texas, USA, sc-166802, 1:200) overnight at 4°C and then with the corresponding secondary antibody for an additional 1 hours at room temperature in dark. Images were captured with Confocal Laser Scanning Microscope (Olympus FV3000, Toyoko, Japan) and the intensities were determined by Image J.

Filipin staining

A fresh stock solution of filipin (Yuanye bio-technology co., Ltd, Shanghai, China) at 5 mg/mL was prepared in ethanol. Then 4 μm-thick frozen sections of intestine were generated, fixed with 4% paraformaldehyde (Servicebio, China), stained with 50 μg/mL filipin for 30 min at room temperature, washed with PBS and then mounted[2]. Images were visualized by confocal laser scanning microscope (Olympus FV3000, Japan) and quantified by Image J.

Fecal cholesterol measurements in mice

Feces were collected for 2 days and dried, followed by ground up prior to analysis as previously described[2]. Dry feces (100 mg) were extracted with 10 ml of methanol: chloroform (1:2, vol/vol) twice, and then the supernatants were collected. Exactly 100 μL of the supernatants was removed and evaporated to dryness. Total cholesterol was dissolved in double distilled water and measured with total cholesterol kit (Nanjingjiancheng, Jiangsu, China).

16S rDNA sequencing and analysis in mice

Cecal DNA was isolated with MagMAX™ Microbiota Ultra Nucleic Acid Isolation Kit, (Thermo Fisher Scientific, USA) and stored at −80°C until analysis by 16S rDNA sequencing at V3–V4 regions on Illumina MiSeq platform (Illunima, San Diego, California, USA) by Novogene Co., Ltd (Beijing, China). Sequencing data for all samples were deposited at NCBI Sequencing Read Archive (SRA Accession: PRJNA887908). MiSeq (Illumina) fastq files were analyzed using Qiime2 (v.2018.4.0). De-noising and amplicon sequencing variants (ASVs) tables were constructed using open-source software package DADA2[3]. ASVs were assigned with taxonomic annotations by applying the feature-classifier plugin (Silva-132-99-nb-classifier) to our representative sequences. The resulting ASV table was filtered to include only ASVs with ≥0.1% relative abundance in at least 50% samples. Significantly altered ASVs were identified using Wilcoxon test in R software version 4.0.3 (R Foundation for Statistical Computing, Vienna, Austria). To deduce the community diversity between the samples, nonmetric multidimensional scaling (NMDS) based on Bray-Curtis distance and *PERMANOVA* based on adonis function were performed by vegan package. Correlation between the phenotypes and ASVs was assessed by Spearman's correlation tests. KEGG ontology profiles, determined by PICRUSt2 (phylogenetic investigation of communities by reconstruction of unobserved states)[4], were assigned to gut metabolic modules using Gomixer[5]. Statistically over/under-represented metabolic modules between mice fed with NCD or HCD were determined by Gomixer with Wilcoxon rank-sum test, and the Benjaminiand Hochberg (*BH*) was used to correct for multiple testing.

Quantification of *Lachnospiraceae* in human fecal samples

The fecal DNA concentrations from both human and mice were measured with Tecan Infinite 200 PRO microplate reader (Infinite M200, Tecan, Switzerland) and loaded at 40 ng for qPCR analysis. The average cycle threshold (Ct) value was calculated from triplicates and duplicates with >2 cycle differences were excluded from the analysis. The relative abundance of *Lachnospiraceae* was calculated as Ct (*Lachnospiraceae*)-Ct (16S ribosome genes), and Ct value for any sample not amplified after 40 cycles was defined as 40 (the threshold of detection). The primer sequences for *Lachnospiraceae* and 16S rRNA (total bacteria) were listed in Supplementary Table 2.

LC-MS based metabolomics profiling in mice

Plasma samples taken at the same day were used for metabolomics profiling by AB Sciex QTRAP 6500 LC-MS/MS platform (MetWare, http://www.metware.cn/). MS spectrometric data was collected by Sciex Q-Trap 6500+ (https://sciex.com.cn/) equipped with an ESI Turbo Ion-Spray interface operating in both positive and negative ion modes and controlled by Analyst 1.6 software (AB Sciex). Metabolites were identified based on HMDB (https://hmdb.ca/). Multivariate statistical analysis method, including orthogonal partial least squares-discriminant analysis (OPLS-DA), was employed to process the metabolites data. Significantly altered metabolites by HCD were determined by variable importance in the projection (VIP ≥1) and absolute Log_2FC (fold change) ≥1.0. Correlations between the phenotypes, significantly altered ASVs and metabolites were assessed by Spearman's correlation tests. A significance level of $p<0.1$ (adjusted *p-value* by *BH*) was adopted for all analyses.

Targeted metabolomics analysis for TMAO, SCFAs, and precursors of 3-MH

Plasma and caecum samples were collected and frozen at -80°C until further analysis. A targeted analysis was carried out by using a HPLC-MS method to quantify TMAO and precursors of 3-MH in plasma samples as described with minor modification[5]. Briefly, plasma samples (30 µL) and standard solutions (10 µL) were added to a mixture of ethylacetate and isopropanol (1:4, v/v, 200 µL) including internal standard, 3-Methyl-d3-L-histidine (3-MH-d3), at 3 µmol/L. After vortex and ultrasonication with ice for 10 min, proteins were precipitated by centrifugation at 18,000g at 4°C for 15 min. Caecum contents (30mg) were homogenized with acetonitrile:methanol (4:1, v/v, 200 µL) for 5 min and incubated at -20℃ for 10 min, before centrifugation at 18,000g at 4°C for 15 min. The supernatant was recovered for further analysis using an Agilent 1290 Infinity II HPLC system (Agilent Technologies, Inc., Santa Clara, CA, USA) interfaced to an Agilent 6495 Triple Quadrupole LC/MS system (Agilent Technologies, Inc. USA) equipped with an AJS. The chemical separation was achieved on a C18 column (Agilent, 2.1 mm ×100 mm, 2.7 µm). The MS was operated in positive ion mode scheduled multiple reaction monitoring modes (MRM). For positive ion analysis, the programmed gradient of solvents was from 5% B to 95% B (A, water + 0.1% formic acid; B, acetonitrile+0.1% formic acid) at 0.3 mL/min. The optimized ion source parameters were as follows: capillary voltage: 3500 V, nozzle voltage: 1500 V. Nitrogen

was applied as a nebulizer gas of 20 psi, a carrier gas of 14 L/min at 200 °C, and a sheath gas of 10 L/min at 350 °C. Analyte-specific mass transitions were as follows: m/z 76→58.2 for TMAO, 170.1→96.1 for 3-MH, 173→99.1 for 3-MH-d3, 241.1→109 for anserine, 90.1→44.2 for alanine, 134.1→88.1 for aspartate, 150.1→56.1 for methionine, 399.1 → 250 for s-adenosyl-l-methionine, 385.1 → 136.1 for S-Adenosylhomocysteine, 136→90.1 for homocysteine. Pooled plasma or caecum reference samples were inserted every 15 samples as an additional quality control. The linearity regression coefficient was R^2>0.99. The inter- and intra-assay coefficients of variation were <10%. The MS raw data were processed by Agilent quantitative software.

As for SCFAs, a targeted analysis was carried out by using a Gas chromatography (GC)-mass spectrometry method as described[6]. Plasma samples (30 μL) and internal standards at 400 μmol/L were added to ethanol (3:1, v/v), then homogenized with hexane (800 μL) for 3 min, and centrifuged at 18,000g at 4°C for 15 min. Lower organic layer was collected and mixed with Hydrochloric acid, and then injected into Agilent HP6890 Series GC System equipped with an Agilent 5973 MS Detector. 1 μL of samples was injected with a 1:8 split ratio on a ZB-WAXplus GC column (30 m × 0.25 mm × 0.25um, Phenomenex), with He as the carrier gas at a flow rate of 1 mL/min. The injector temperature was 260 °C, and the column temperature was isocratic at 300 °C. SCFAs were normalized to the internal standards by creating a linear calibration curve with accuracy higher than 80% for each standard.

Cholesterol uptake and transport in Caco-2 cells

Caco-2 cells were seeded in a 12-well plate containing 4×10^5 cells per well for the measurement of cholesterol uptake. Cells pre-treated with vehicle control or 3-MH were further incubated with NBD-cholesterol (Thermo Fisher Scientific, USA) at 10 μM for 8 h. After incubation, cells were washed with PBS containing 5mM Taurocholic acid sodium salt twice and lysed using RIPA. Cell lysates were measured for fluorescence at excitation 485nm and emission 535nm. For experiments concerning the transport of cholesterol, Caco-2 cells were seeded on the filter insert membrane (Polyester membrane, 0.4 μM, Labselect, Hefei, China) as previously described[7]. Fully polarized cells were replaced with new medium with or without 3-MH and incubated for another 12h before analysis. The final lipid micelles preparation consisted of serum-

free medium with 2 mM Taurocholate (Sigma Aldrich), 0.6 mM oleic acid (Sigma Aldrich), 0.2 mM L-α-phosphatidylcholine (Sigma Aldrich), 0.2 mM L-α-lysophosphatidylcholine (Sigma Aldrich), 0.2 mM 2-monooleoylglycerol (Sigma Aldrich), and 10 μM NBD-cholesterol (Thermo Fisher Scientific). After pretreatment with 3-MH, the apical medium was replaced with 0.4 mL NBD-cholesterol micelles solution or MEM medium without phenol red and the basolateral medium was replaced with 1.2 mL NBD-cholesterol micelles solution or MEM medium without phenol red. After 0, 30, 60, 90, and 120 min after incubation, samples from the receiving compartment (100 μL) were collected and replaced by an equal volume of the MEM medium and then subjected to fluorescence counting analysis. The apparent permeability coefficients P_{app} (cm/s) were calculated according to the following equation[8]: $P_{app}=(dQ/dt) \cdot V_A/(c_0 \cdot A)$, where dQ/dt was the change in concentration on the receiving side within a given time period, V_A was the volume of the receiving chamber, c_0 was the initial concentration of the tested compounds and A was the surface area of the monolayer (1.1 cm^2).

siRNA-mediated gene silencing

To knockdown genes of interest in Caco-2 cells, siRNAs targeting human NPC1L1 and HNF1A were designed and synthesized by Ribobio (Guangdong, China). The sequences of siRNAs targeting human NPC1L1 and HNF1A were 5′-AAGGCATGAAGGAATCACAGA-3′ and 5′-AAGCAAGTGTACGACGCGCAC -3′, respectively. Caco-2 cells were transfected with siRNAs using ribo*FECT* CP transfection (Ribobio, China) following the manufacturer's instructions. Cells were collected after 48 h of transfection to examine the knock-down efficiency. The nonspecific siRNA control (NC siRNA) was used as a negative control.

Luciferase reporter assay

The NPC1L1 luciferase reporter plasmids and control plasmids were purchased from Wuhan GeneCreate Biological Engineering Co., Ltd. For the analysis of promoter activities and transcriptional regulation of NPC1L1, the 2 kb promoter region of NPC1L1 gene or mutant (-1914/-1928) site was cloned in pGL3 basic vector using the restriction enzymes *Kpn*I and *Xho*I. Plasmids were transfected into Caco-2 cells using lipo3000 transfection system (Thermo fisher scientific, USA). After 36 h of transfection,

Caco-2 cells were further incubated with or without 3-MH at 20 μM for another 12 h before analysis. Then, luciferase and *Renilla* were determined using the dual-luciferase reporter assay system (E1910, Promega, Shanghai, China) according to the manufacturer's instructions.

Chromatin immunoprecipitation assay (ChIP)

ChIP assay was performed according to the manufacturer's instruction (Thermo fisher scientific, USA). Briefly, about 100mg of frozen jejunum samples from mice gavaged with PBS or 3-MH for 12 weeks were grounded in liquid nitrogen and kept on dry ice. Jejunum and Caco-2 cell samples were cross-linked with 1% formaldehyde, rotated for 20min, followed by adding 0.125 mol/L glycine solution to stop the cross-linking. Then samples were centrifuged and washed with ice-cold PBS twice before homogenization. After lysis and sonication, samples were incubated overnight at 4°C with anti-HNF1A antibody (GeneTex, GTX113850, USA) or rabbit anti-IgG antibody as negative control. Immunocomplexes were then collected with magnetic beads and incubated for 1.5 h at 65°C in high-salt solution to reverse the crosslink reaction. DNA fragments were analyzed by real-time PCR with the following primer pair for human NPC1L1 promoter: 5'-CTGGTATCACTGGAAGCGAGT-3' and 5'-CACGCGGGTCACATTGATGA-3', and mouse NPC1L1 promotor: 5'-CGCCCTTCTTTCTACATGGGT-3' and 5'- GAATCTGCGCTTACGAGGGAG-3', respectively. The resulting products were separated on 1.5% agarose gel and stained with GelRed, while 10% input of DNA solution was analyzed simultaneously for normalization.

Real-time quantitative RT-PCR

Total RNA was extracted from tissues or cells by EastepTM Super Total RNA Extraction Kit (LS1040, Promega, China). Total RNA of each sample (500 ng) was reversely transcribed to cDNA using Transcriptor First Strand cDNA Synthesis Kit (Takara Bio). The quantitative real-time PCR was performed to determine the mRNA expression using PowerUp SYBR Green assay (Applied Biosystems, California, USA). The specific primers used in this study were shown in Table S2.

Western blotting

Total protein (30 μg) was extracted and separated on 10% SDS-PAGE, before being transferred onto polyvinylidene fluoride (PVDF) membranes (Millipore Corp, Billerica, MA, USA). After blocking with 5% milk (w/v) in Tween-20/Tris-buffered saline (TBST) at room temperature for 2h, and bolts were incubated with various primary antibodies, including NPC1L1 (Santa Cruz, sc-166802, 1:1000), HNF1A (GeneTex, CA, USA, GTX113850, 1:1000), β-actin (Santa Cruz, sc47778, 1:1000) and GAPDH (Proteintech, Wuhan, China, 60004-1-Ig, 1:80000), followed by HRP-conjugated secondary antibody at room temperature for another 1.5h. The specific protein bands were detected with an enhanced chemiluminescence (ECL, Thermo Fisher Scientific, USA).

Supplementary references

1. Sparrow CP, Patel S, Baffic J et al. A fluorescent cholesterol analog traces cholesterol absorption in hamsters and is esterified in vivo and in vitro. *Journal of Lipid Research*. 1999; 40(10):1747-57.
2. Zhang YY, Fu ZY, Wei J et al. A LIMA1 variant promotes low plasma LDL cholesterol and decreases intestinal cholesterol absorption. *Science*. 2018; 360(6393):1087-92.
3. Callahan BJ, McMurdie PJ, Rosen MJ et al. DADA2: High-resolution sample inference from Illumina amplicon data. *Nat Methods*. 2016;13(7):581-3.
4. Douglas GM, Maffei VJ, Zaneveld JR et al. PICRUSt2 for prediction of metagenome functions. *Nat Biotechnol*. 2020;38(6):685-8.
5. Wang Z, Bergeron N, Levison BS et al. Impact of chronic dietary red meat, white meat, or non-meat protein on trimethylamine N-oxide metabolism and renal excretion in healthy men and women. *Eur Heart J.* 2019; 40, 583-594.
6. Han X, Guo J, You Y et al. A fast and accurate way to determine short chain fatty acids in mouse feces based on GC-MS. *J Chromatogr B Analyt Technol Biomed Life Sci*. 2018; 1099, 73-82.
7. Darzi Y, Falony G, Vieira-Silva S et al. Towards biome-specific analysis of meta-omics data. *ISME J*. 2016; 10(5):1025-8.
8. Hiebl V, Schachner D, Ladurner A et al. Caco-2 Cells for Measuring Intestinal Cholesterol Transport-Possibilities and Limitations. *Biol Proced Online*. 2020; 22:7.
9. Krishna G, Chen K, Lin C et al. Permeability of lipophilic compounds in drug discovery using in-vitro human absorption model, Caco-2. *Int J Pharm*. 2001; 222(1):77-89.

Supplementary Figures

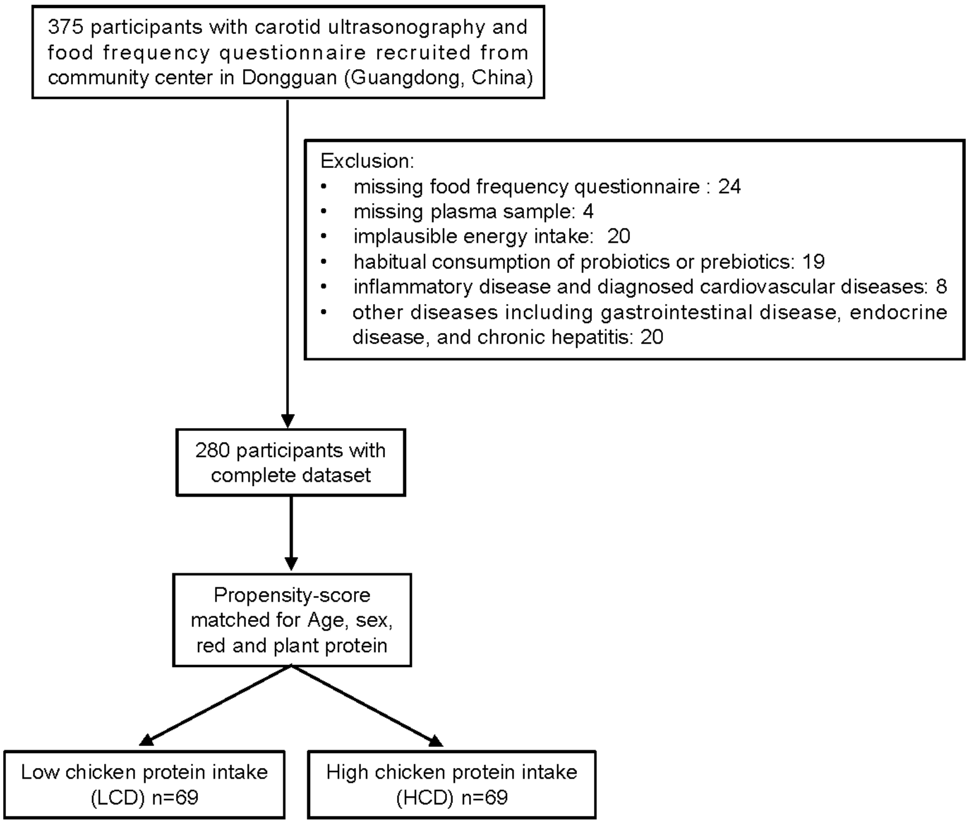

Figure S1. Flow chart of the study participants.

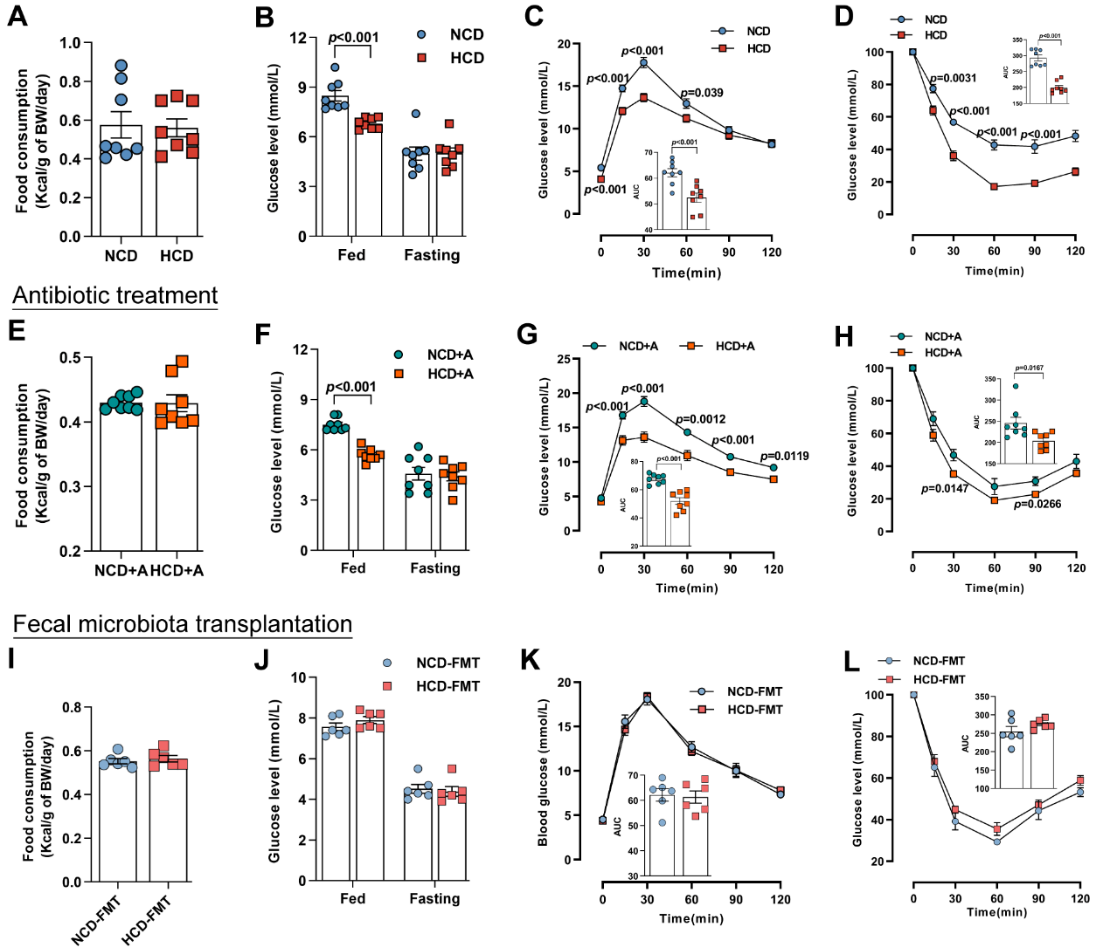

Figure S2. Glucose metabolism was improved by HCD independent of gut microbiota. (A-D) Mice were fed with NCD or HCD for 12 weeks before analysis (n=8 per group). (E-H) Mice fed with NCD or HCD were exposed to antibiotics cocktail for 12 weeks before analysis (n=8 per group). (I-L) Mice fed with normal chow received fecal microbial transplantation from donors fed with NCD or HCD for 12 weeks before analysis (n=6 per group). (A, E, I) Food intake during intervention. (B, F, J) Glucose level at both fasting and fed status. (C, G, K) Glucose tolerance test. (D, H, L) Insulin tolerance test. Data were presented as mean ± SEM. Statistical analysis was performed by unpaired *t* test.

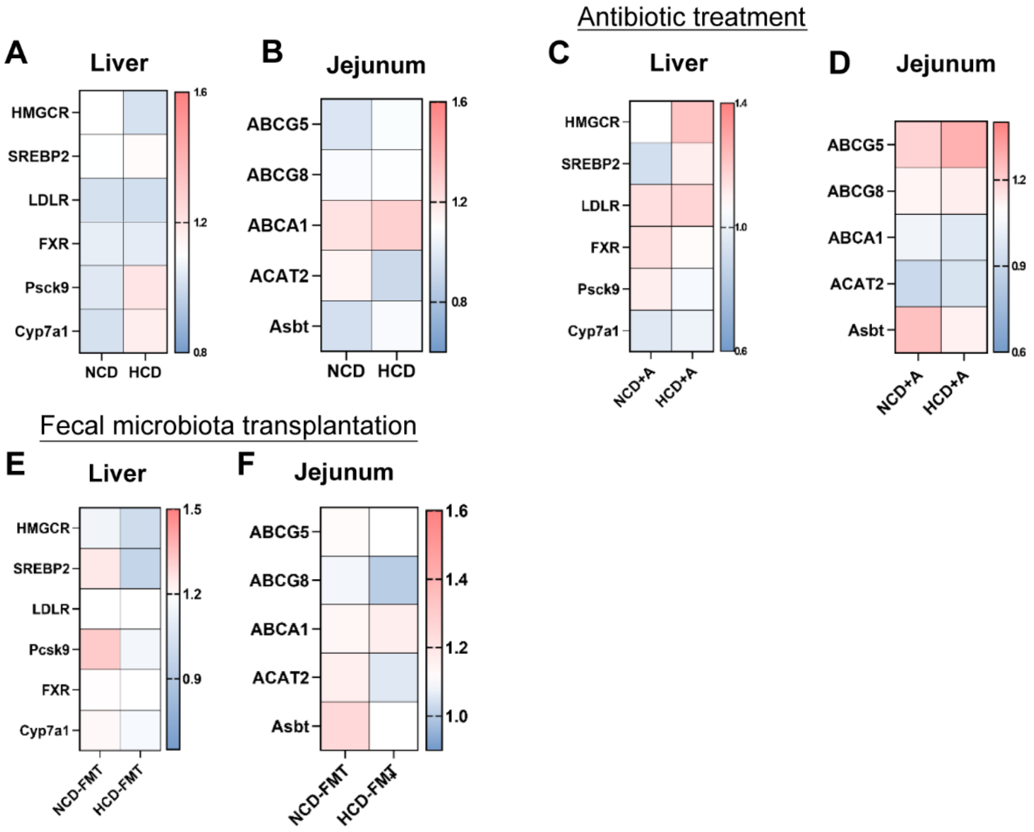

Figure S3. Expression of genes involved in cholesterol biosynthesis and lipid transport. Mice were fed with (A, B) NCD or HCD, (C, D) exposed to antibiotics cocktail, or (E, F) colonized with fecal microbiota from donors fed with NCD or HCD for 12 weeks before liver and intestine were harvested and subjected to real-time PCR analysis. (A, C, E) Expression of genes involved in hepatic cholesterol biosynthesis and transport. (B, D, F) Expression of genes involved in intestinal cholesterol metabolism under different treatments. Statistical analysis was performed using unpaired *t* test. Data were presented as mean ± SEM. (n=8 per group for A-D, and n=6 per group for E, F).

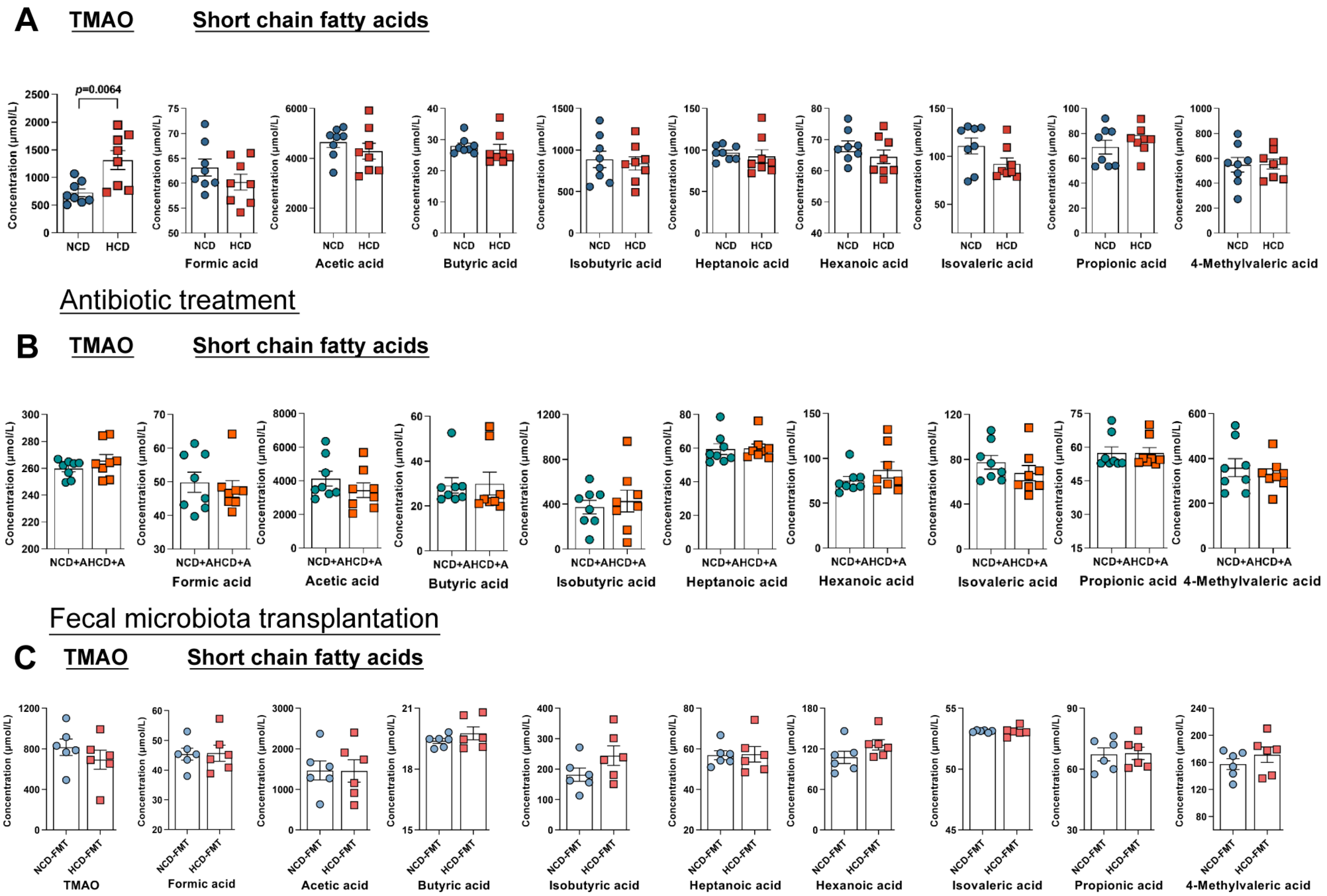

A
TMAO
Short chain fatty acids
p=0.0064
Concentration (μmol/L)
NCD
HCD
Formic acid
Acetic acid
Butyric acid
Isobutyric acid
Heptanoic acid
Hexanoic acid
Isovaleric acid
Propionic acid
4-Methylvaleric acid
Antibiotic treatment
B
TMAO
Short chain fatty acids
Concentration (μmol/L)
NCD+A
HCD+A
Formic acid
Acetic acid
Butyric acid
Isobutyric acid
Heptanoic acid
Hexanoic acid
Isovaleric acid
Propionic acid
4-Methylvaleric acid
Fecal microbiota transplantation
C
TMAO
Short chain fatty acids
Concentration (μmol/L)
NCD-FMT
HCD-FMT
TMAO
Formic acid
Acetic acid
Butyric acid
Isobutyric acid
Heptanoic acid
Hexanoic acid
Isovaleric acid
Propionic acid
4-Methylvaleric acid

Figure S4. Plasma levels of TMAO and SCFAs. Plasma levels of TMAO and various kinds of SCFAs, including formic acid, acetic acid, butyric acid, isobutyric acid, hexanoic acid, heptanoic acid, isovaleric acid, propionic acid and 4-methylvaleric acid, were determined in (A) mice fed with NCD or HCD for 12 weeks (n=8), (B) NCD or HCD-fed mice exposed to antibiotics cocktail for 12 weeks (n=8), and (C) NCD-fed mice receiving fecal microbial transplantation from donors challenged with NCD or HCD for 12 weeks (n=6). Data were presented as mean±SEM. Statistical analysis was performed by unpaired *t* test.

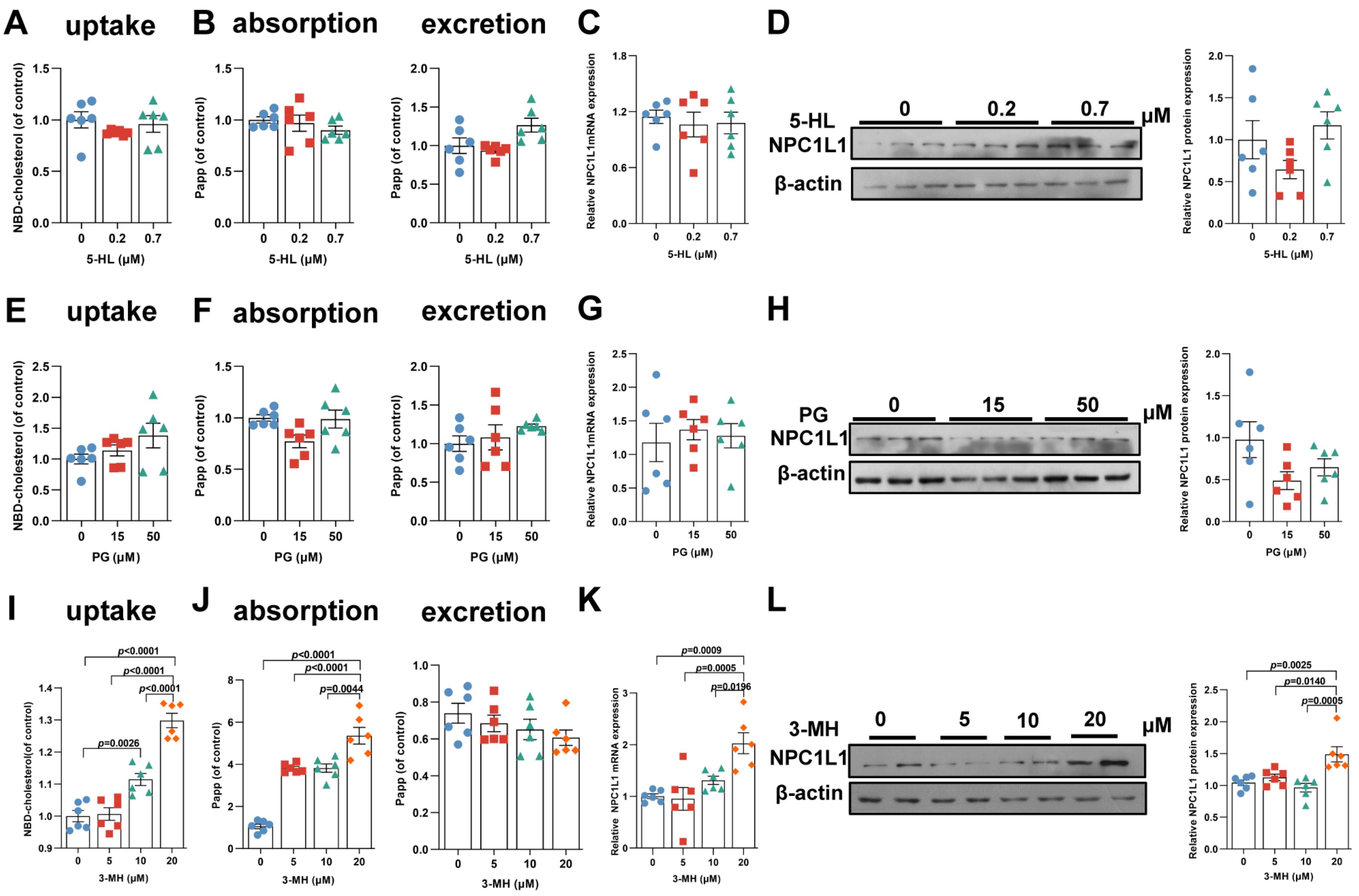
A uptake
B absorption
excretion
C
D
NBD-cholesterol (of control)
Papp (of control)
Relative NPC1L1mRNA expression
Relative NPC1L1 protein expression
5-HL (μM)
5-HL
NPC1L1
β-actin
0
0.2
0.7
μM
E uptake
F absorption
excretion
G
H
PG (μM)
PG
0
15
50
I uptake
J absorption
excretion
K
L
3-MH (μM)
3-MH
0
5
10
20
p<0.0001
p<0.0001
p<0.0001
p=0.0026
p<0.0001
p<0.0001
p=0.0044
p=0.0009
p=0.0005
p=0.0196
p=0.0025
p=0.0140
p=0.0005

Figure S5. The effect of top 3 selected metabolites on cholesterol absorption in Caco-2 cells. Caco-2 cells were treated with various dosages of (A-D) 5-HL, (E-H) PG, or (I-L) 3-MH as indicated before analysis. (A, E, I) Cholesterol uptake. (B, F, J) Cholesterol absorption and excretion. Expression of NPC1L1 at both (C, G, K) mRNA, and (D, H, L) protein levels. Data were expressed as mean ± SEM (n=6 independent experiments). Statistical analysis was performed by one-way ANOVA followed by Tukey's test.

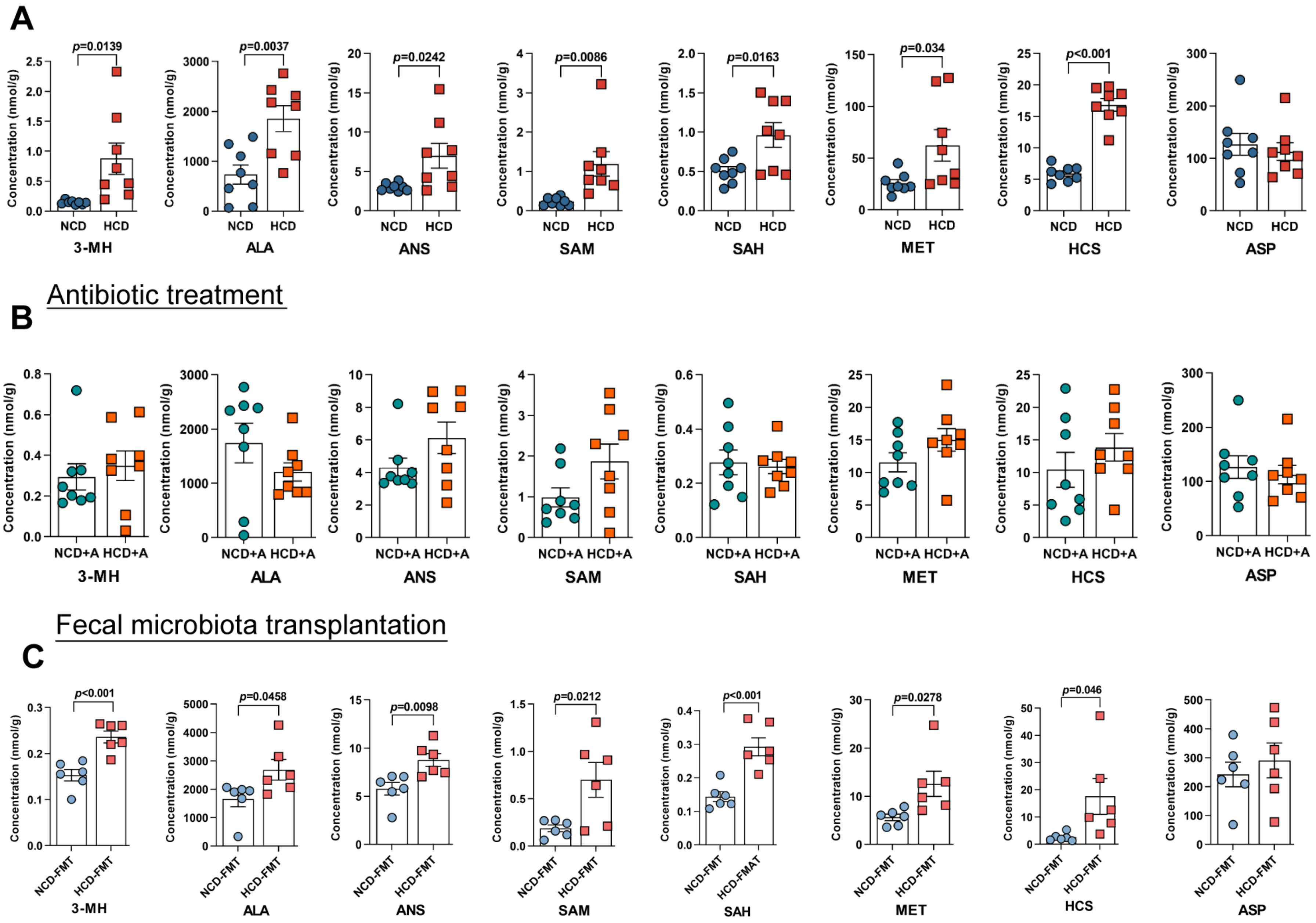
A
p=0.0139
p=0.0037
p=0.0242
p=0.0086
p=0.0163
p=0.034
p<0.001
Concentration (nmol/g)
NCD HCD
3-MH
ALA
ANS
SAM
SAH
MET
HCS
ASP
Antibiotic treatment
B
NCD+A HCD+A
3-MH
ALA
ANS
SAM
SAH
MET
HCS
ASP
Fecal microbiota transplantation
C
p<0.001
p=0.0458
p=0.0098
p=0.0212
p<0.001
p=0.0278
p=0.046
NCD-FMT
HCD-FMT
HCD-FMAT
3-MH
ALA
ANS
SAM
SAH
MET
HCS
ASP

Figure S6. Abundance of precursors of 3-MH in caecum contents. Abundance of intermediate metabolites or precursors along the pathways responsible for 3-MH production, including alanine (ALA), anserine (ANS), *S*-adenosyl-L-methionine (SAM), S-adenosyl-L-Homocysteine (SAH), methionine (MET), homocysteine (HCS) and aspartate (ASP), in (**A**) mice fed with NCD or HCD for 12 weeks (n=8), (**B**) NCD or HCD-fed mice exposed to antibiotics cocktail for 12 weeks (n=8), and (**C**) NCD-fed mice receiving fecal microbial transplantation from donors challenged with NCD or HCD for 12 weeks (n=6). Data were presented as mean±SEM. Statistical analysis was performed by unpaired *t* test.

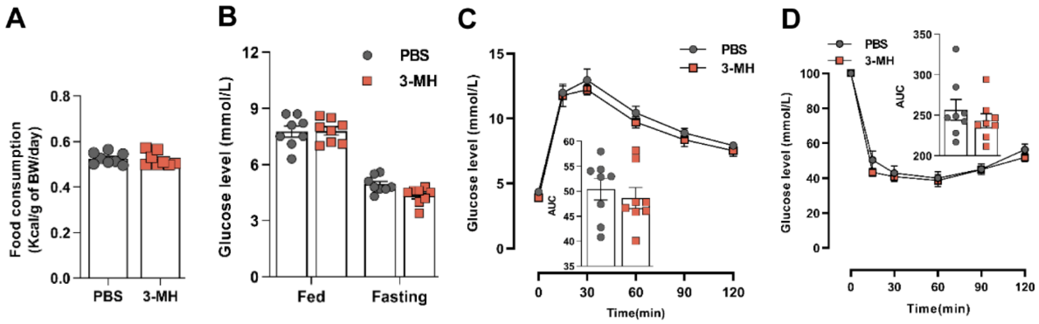

Figure S7. 3-MH exerted no significant effect on glucose metabolism. ApoE$^{-/-}$ mice were gavaged with 3-MH or PBS control for 12 weeks before analysis. (A) Food intake during intervention. (B) Glucose level at both fasting and fed status. (C) Glucose tolerance test. (D) Insulin tolerance test. Data were presented as mean ± SEM (n=8 per group). Statistical analysis was performed by unpaired *t* test.

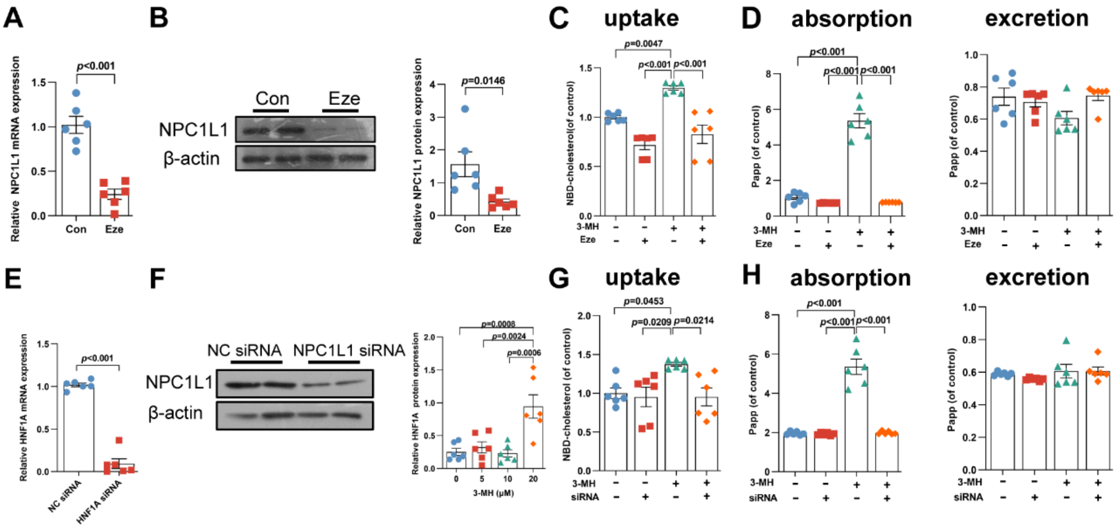

Figure S8. Inhibition of NPC1L1 abolished the effect of 3-MH on cholesterol absorption in Caco-2 cells. Caco-2 cells were pre-treated with (A-D) Ezetimibe, NPC1L1-specific inhibitor, or (E-H) specific siRNA targeting NPC1L1 before further analysis. NPC1L1 expression at both (A, E) mRNA and (B, F) protein levels. (C, G) cholesterol uptake, and (D, H) cholesterol absorption and excretion. Data were expressed as mean ± SEM (n=6 independent experiments). Statistical analysis was performed by unpaired *t* test for A, B, E and F, and one-way ANOVA followed by Tukey's test for C, D, G and H.

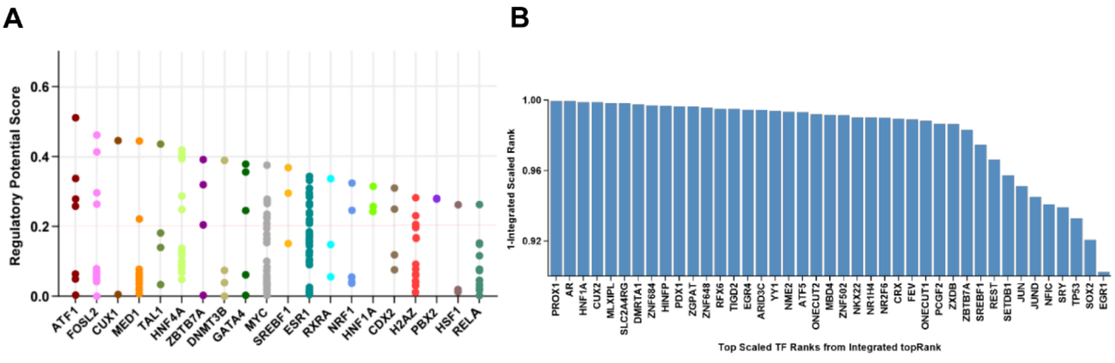

Figure S9. *In silico* analysis of potential transcription factors regulating NPC1L1.

(A) Potential regulators of NPC1L1 transcription derived from Cistrome DB Toolkit.

(B) Potential regulators of NPC1L1 transcription derived from ChEA3 Database.

Supplementary Tables

Table S1. Macronutrient composition and calories of NCD and HCD.

	NCD	HCD
**Macronutrients**		
Protein (% Kcal)	15	30
Fat (% Kcal)	30	30
Carbohydrate (% Kcal)	55	40
Caloric profile (Kcal/g)	4.364	4.364
**Ingredient (g/100g)**		
Chicken protein powder	17.1	37.1
Corn Starch	13.93	11.51
Maltodextrin	9.98	5.98
Sucrose	34.11	25.11
Cellulose	4.99	3.48
Corn oil	0.99	0.99
Anhydrous cream	13.55	10.48

Table S2. Primer sequences for qRT-PCR.

Species	Gene	Sequence (5'→3')	
**Mouse**	*HMGCR*	F: CTGGAATTATGAGTGCCCCAAA	R: ACGACTGTACTGAAGACAAAGC
	SREBP2	F: CAGGTGCAGACGGTACAGG	R: CGACCCTTACTGGCACTTGAA
	LDLR	F: TGACTCAGACGAACAAGGCTG	R: ATCTAGGCAATCTCGGTCTCC
	Psck9	F: GAGACCCAGAGGCTACAGATT	R: AATGTACTCCACATGGGGCAA
	FXR	F: GCTTGATGTGCTACAAAAGCTG	R: CGTGGTGATGGTTGAATGTCC
	Cyp7a1	F: GGGATTGCTGTGGTAGTGAGC	R: GGTATGGAATCAACCCGTTGTC
	NPC1L1	F: TGTCCCCGCCTATACAATGG	R: CCTTGGTGATAGACAGGCTACTG
	ABCG5	F: CGTGGCGGACCAAATGATT	R: CCACTGGAAATTCCCCCAAA
	ABCG8	F: CTGTGGAATGGGACTGTACTTC	R: GTTGGACTGACCACTGTAGGT
	ABCA1	F: AAAACCGCAGACATCCTTCAG	R: CATACCGAAACTCGTTCACCC
	ACAT2	F: CCCGTGGTCATCGTCTCAG	R: GGACAGGGCACCATTGAAGG
	Asbt	F: GTCTGTCCCCCAAATGCAACT	R: CACCCCATAGAAAACATCACCA
	β-actin	F: CCCCACTCCTAAGAGGAGGAT	R: CTCAGACCTGGGCCATTCAG
**Human**	*NPC1L1*	F: CTGGTATCACTGGAAGCGAGT	R: CACGCGGGTCACATTGATGA
	ZBTB7A	F: GCTTGGGCCGGTTGAATGTA	R: GGCTGTGAAGTTACCGTCGG
	SREBF1	F: GCCCCTGTAACGACCACTG	R: CAGCGAGTCTGCCTTGATG
	HNF1A	F: AACACCTCAACAAGGGCACTC	R: CCCCACTTGAAACGGTTCCT
	β-actin	F: CATGTACGTTGCTATCCAGGC	R: CTCCTTAATGTCACGCACGAT
**Bacteria**	*Total bacteria*	F: GGTGAATACGTTCCCGG	R: TACGGCTACCTTGTTACGACTT
	Lachnospiraceae	F: GCAGTGGGGAATATTGCA	R: CTTTGAGTTTCATTCTTGCGAA
